# Molecular crosstalk in perivascular adipose tissue: mechanisms of inflammation, metabolic dysregulation, and therapeutic opportunities in cardiovascular disease

**DOI:** 10.3389/fcvm.2025.1613900

**Published:** 2025-06-26

**Authors:** Shunhong Zhang, Jun Jiang, Yi Luo, Guangyue Liu, Saidi Hu, Siran Wan, Chenchen Luo, Hong Li, Nian Li, Jailson da Graça Espírito Santo Vasconcelos, Leonilde Lavres Ceita de Carvalho, Monazeri Lima Bragança da Costa, José Etchu Takounjou, Karem Maimite Das Neves, Luzimery dos Ramos da Conceição, Marinela da Costa Encarnação, Lin-Yong Zhao

**Affiliations:** ^1^ Department of Cardiology, Pangang Group General Hospital, Panzhihua, China; ^2^ Department of Anesthesiology, West China Hospital, Sichuan University, Chengdu, China; ^3^ Department of Stomatology, Yaan People’s Hospital, Yaan, China; ^4^ Department of Gynaecology and Obstetrics, Yaan People’s Hospital, Yaan, China; ^5^ Department of Outpatient Chengbei, The Affiliated Stomatological Hospital, Southwest Medical University, Luzhou, China; ^6^ Department of Ultrasound Medicine, Panzhihua Women & Enfants Healthcare Hospital, Panzhihua, China; ^7^ Department of Traditional Chinese Medicine, Panzhihua Central Hospital, Panzhihua, China; ^8^ Department of General Surgery, Hospital Dr. Ayres de Menezes, Sao Tome, Sao Tome and Principe; ^9^ Department of Stomatology, Delegation of Health of Sao Tome and Principe, Sao Tome, Sao Tome and Principe; ^10^ Department of General Surgery & Laboratory of Gastric Cancer, State Key Laboratory of Biotherapy/Collaborative Innovation Center of Biotherapy and Cancer Center, West China Hospital, Sichuan University, Chengdu, China

**Keywords:** perivascular adipose tissue, biomarkers, vascular inflammation, atherosclerosis, cardiovascular disease

## Abstract

The escalating recognition of perivascular adipose tissue (PVAT) as a molecular nexus in cardiovascular disease (CVD) pathogenesis necessitates a comprehensive synthesis of its spatiotemporal dynamics and therapeutic potential. This review synthesizes PVAT's roles in vascular inflammation, metabolic dysregulation, and emerging diagnostic strategies, emphasizing molecular cross-talk and spatial heterogeneity. We explore PVAT's molecular interactions in obesity, diabetes, and hypertension, elucidating its contribution to inflammation, oxidative stress, and endothelial dysfunction. Advanced imaging techniques, notably the perivascular fat attenuation index (FAI) and circulating biomarkers, are highlighted for early CVD detection. Novel therapeutic strategies, including lifestyle modifications, pharmacological interventions, and gut microbiota modulation, are discussed. Finally, we emphasize multi-omics approaches and propose a roadmap bridging basic and clinical research to advance PVAT-based CVD management.

## 1 Introduction

Perivascular adipose tissue (PVAT) is of paramount importance as it closely surrounds blood vessels and releases a variety of active substances through paracrine and autocrine mechanisms (1, 2). PVAT is essential in the complex process of regulating vascular tension, which is vital for the proper functioning of the cardiovascular system. By maintaining the elasticity of blood vessels, PVAT ensures that they can efficiently accommodate the flow of blood, adapting to changes in pressure and volume (3). Furthermore, PVAT is involved in the intricate processes of lipid and energy metabolism, highlighting its importance in the body's overall metabolic functions (4). In addition to these roles, PVAT also plays a significant part in regulating inflammation and immune responses, demonstrating its multifaceted contributions to both vascular health and systemic metabolism (5).

Conversely, under pathological conditions such as obesity and diabetes, PVAT function becomes impaired. The secretion of active substances becomes unbalanced, leading to excessive release of inflammatory factors and disordered immune regulation (6, 7). This dysfunction contributes to impaired vascular endothelial function, accelerated atherosclerosis, and a series of vascular pathologies, thereby becoming a significant driving factor in the onset and progression of cardiovascular diseases (CVDs), which profoundly impacts overall health and disease processes (8–10). The recently discovered role of PVAT as a modulator of circadian rhythms—via Bmal1-dependent secretion of heme-binding protein—links nocturnal blood pressure dipping to adipose redox cycles (11), thereby offering mechanistic insights into cardiovascular events related to morning surges.

Although the significance of PVAT in both cardiovascular physiological and pathological processes has gained increasing recognition; however, current research in this area continues to encounter several limitations. This review challenges three prevailing assumptions: (1) Homogeneity Fallacy: PVAT is not a monolithic entity; rather, it represents a mosaic of adipocyte subtypes and immune niches characterized by distinct spatial metabolic programming. (2) Paracrine-Centric View: Mechanotransduction through the extracellular matrix-integrin signaling of PVAT may be as significant as soluble factors in contributing to vascular stiffening. (3) Therapeutic Neutrality: The PVAT-specific effects of current anti-diabetic drugs remain unquantified, which poses a risk of off-target consequences. By integrating single-cell omics, advanced imaging techniques, and data from interventional trials, we propose a novel framework for PVAT-centric cardiovascular therapeutics—one that emphasizes depot-specific targeting, optimization of circadian rhythms, and modulation of the microbiome-adipose axis.

## 2 Paracrine effects and bi-directional cross-talk of PVAT in cardiovascular function

### 2.1 Anatomical location and cellular composition

Anatomically, PVAT surrounds most large blood vessels, excluding the pulmonary and brain vasculature and the microcirculation (12, 13). PVAT is specifically designated as adipose tissue that resides within a radial distance equivalent to the vessel's own width, measured from the outer wall of a blood vessel. When dealing with vessels exceeding a diameter of 2 cm, notably the aorta, PVAT can expand up to a maximum of 2 cm away from the vessel wall (1). This tissue is intimately associated with blood vessels, forming a distinct layer encircling the adventitia. Its distribution patterns vary considerably, contingent upon the vessel's location and size. Notably, in larger arteries like the aorta, PVAT can constitute a substantial depot, whereas in smaller vessels, it may manifest as a thinner, more delicate layer. In rodents, the PVAT surrounding the thoracic aorta consists of brown adipose tissue (BAT), whereas the PVAT around the abdominal aorta is made up of a combination of white adipose tissue (WAT) and a portion of BAT (14, 15). In humans, the phenotype of thoracic aortic PVAT is characterized as brown. In the case of neonates and during the early developmental stages, human epicardial adipose tissue (EAT) exhibits both morphological and functional characteristics that closely resemble those of BAT. This similarity is particularly significant in the context of early life. However, under typical physiological conditions, the brown fat-like attributes of EAT diminish considerably as a person ages, transitioning from childhood into adulthood. This decline highlights the dynamic nature of PVAT and its changing roles throughout the life course (16, 17).

**Figure 1:**
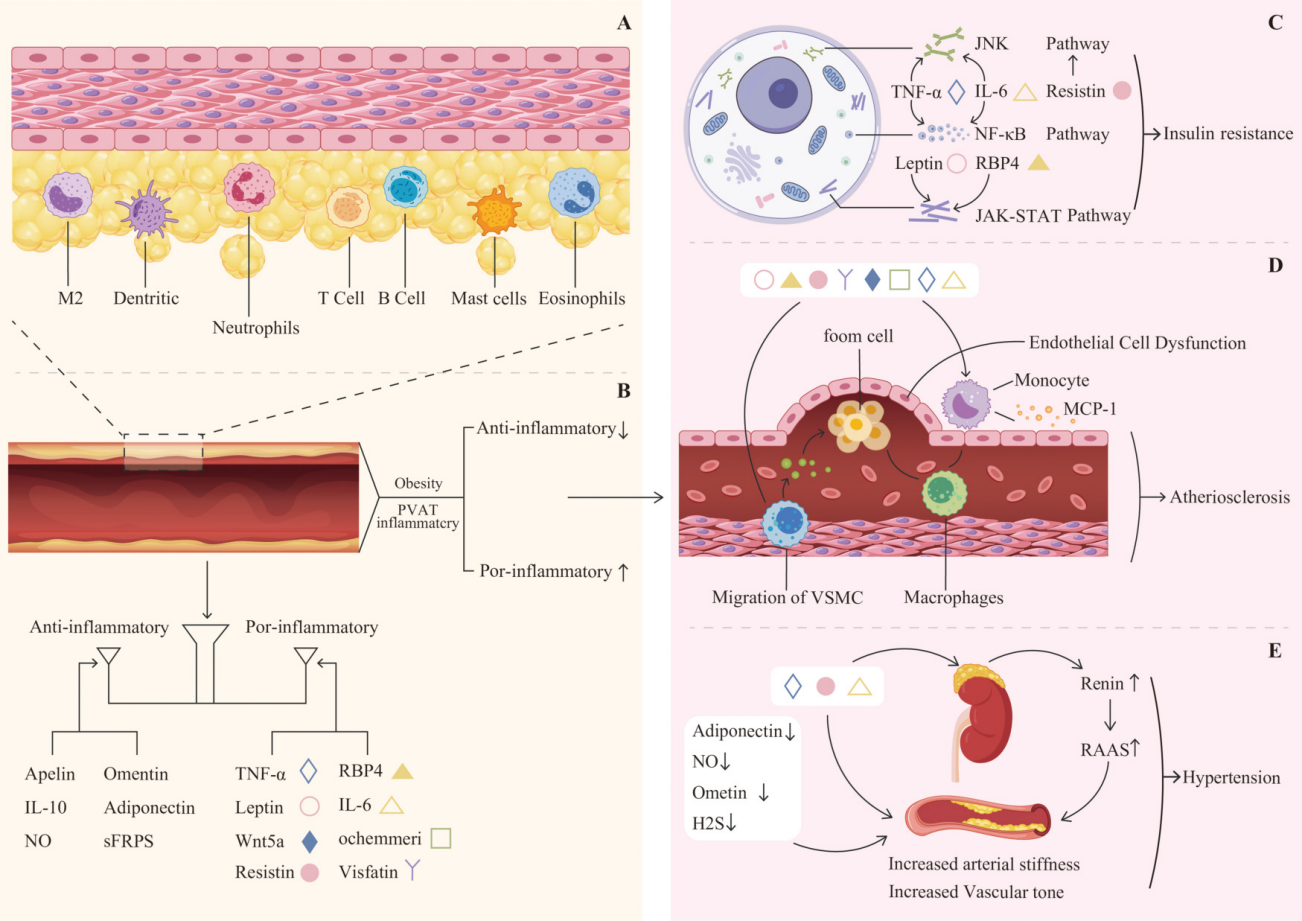
**(A)** The cellular composition of perivascular adipose tissue (PVAT) includes adipocytes, macrophages(M2), neutrophils, dendritic cells, mast cells, eosinophils, T cells, B cells, and various other cell types. **(B)** Balance of anti-inflammatory and pro-inflammatory adipokines. **(C)** Leptin, resistin, RBP4, TNF-αand IL-6 can activate the JNK, NF-κB, and JAK-STAT pathways, leading to insulin resistance. **(D)** Pro-inflammatory factors contribute to endothelial cell dysfunction, as well as the migration of vascular smooth muscle cells and monocytes, ultimately leading to atherosclerosis. **(E)** The reduction of adiponectin, Ometin, and H2S, alongside the increase in TNF-α, IL-6, and resistin, contributes to increased arterial stiffness and vascular tone. This biochemical imbalance promotes the secretion of renin and activates the RAS system, ultimately resulting in hypertension.

Cellularly, PVAT harbors adipocytes, macrophages, neutrophils, dendritic cells, mast cells, eosinophils, T cells, B cells, and various other cell types. These cells interact to maintain the functionality and microenvironmental homeostasis of PVAT (18) (Figure 1A). The adipocytes can be white, brown, or beige, each with unique characteristics and functions, BAT and beige fat function differently than WAT (Table 1). While WAT mainly acts as an energy reservoir, accumulating surplus chemical energy in the form of triacylglycerol (TAG), both BAT and beige fat are noted for their elevated metabolic activity, employing chemical energy for the generation of heat (24). The thermogenic capacity of BAT and beige fat plays an essential role in sustaining body temperature regulation, achieving energy equilibrium, and managing body weight (25). PVAT's role transcends its anatomical proximity to vasculature, functioning as a dynamic signaling interface with depot-specific molecular signatures.

**Table 1:** Distribution and functionality of white, beige, and brown adipocytes.

Comparison items	White adipocytes	Beige adipocytes	Brown adipocytes
Morphological features	Contain a large lipid droplet occupying most of the cell volume, with a flattened nucleus in a “half-moon” shape (19)	Have some small lipid droplets, more mitochondria, and the nucleus is in the center of the cell (19)	Have multiple small lipid droplets, abundant and large mitochondria, and a round nucleus in the center of the cell (19)
Distribution location	Widely distributed in subcutaneous tissue and around internal organs	Mainly in subcutaneous WAT and a small amount in other parts	Mainly in neck, shoulders, clavicle, around spine, between scapulae, adrenal glands, etc.
Main function	Store energy	Have both energy storage and thermogenic functions, and can be activated for thermogenesis under specific conditions	Generate heat by oxidizing fatty acids and other substances to maintain body temperature balance and consume energy
Thermogenic-related proteins	Basically do not express UCP1 (20)	Contain UCP1 but usually with low expression and can be highly expressed under specific stimuli (21)	Highly express UCP1 (21)
Impact on health	Excessive amount can lead to obesity and related chronic diseases (22)	Activation can help improve obesity and related metabolic disorders	Beneficial to health, can stabilize blood sugar and enhance insulin sensitivity, etc. (23)
Content in human body	Most abundant.	Between white adipocytes and brown adipocytes	Less

Recent single-nucleus RNA sequencing (snRNA-seq) analyses reveal three distinct adipocyte subpopulations in human thoracic PVAT: uncoupling protein 1(UCP1) thermogenic, ADIPOQ endocrine, and fibro-inflammatory adipocytes—each exhibiting unique vascular crosstalk patterns (24, 26–30). Notably, In murine models, periaortic PVAT demonstrates higher mitochondrial density compared to mesenteric depots, correlating with enhanced fatty acid oxidation and reactive oxygen species (ROS) buffering capacity (31). This spatial metabolic specialization challenges the traditional dichotomy between white and brown adipocytes, suggesting that PVAT exists as a continuum of plastic phenotypes. Furthermore, it contests the conventional viewpoint of the “Homogeneity Fallacy” regarding PVAT.

### 2.2 PVAT in normal vascular physiology: paracrine and endocrine effects

PVAT releases a variety of bioactive molecules, including adipokines (32), cytokines (33), and growth factors (34). Studies conducted in animal models have demonstrated that these molecules can influence vascular function through endocrine or paracrine mechanisms. Under normal physiological circumstances, PVAT has a net effect of vasodilation, antioxidation, and anti-inflammation on the blood vessels. The vasodilatory function is influenced by the secretion of various factors from adipocytes, which are specialized cells in adipose tissue. Among these factors are adiponectin (35), apelin (36), leptin (37), and omentin (38), each playing a significant role in the regulation of vascular tone. These bioactive compounds contribute to vasodilation through two primary mechanisms. Secondly, they may indirectly promote the production of endothelium-derived vasodilators, including nitric oxide (NO) and the endothelium-derived hyperpolarizing factor. This results in enhanced blood circulation and improved vascular performance. Animal experiments have confirmed this effect (39). Interestingly, differences have been observed among various ethnic groups (40). This intricate interplay underscores the importance of adipocyte-derived factors in maintaining vascular health and regulating blood pressure. The paracrine functions of adipocytokines released from PVAT exhibit significant antioxidant and anti-inflammatory properties. These effects may be mediated by activating the 5′adenosine monophosphate-activated protein kinase (AMPK) and NO signaling pathways in nearby blood vessels. In mouse experiments, the downregulation of AMPK inhibits the synthesis of NO, which lead to a reduction in the anti-contraction activity PVAT and a decrease in the BAT phenotype (31). This interaction suggests that the release of adipokines from PVAT not only influences the local vascular environment but also plays a crucial role in modulating vascular health. By engaging these signaling mechanisms, adipokines contribute to the maintenance of vascular homeostasis, potentially offering protective benefits against inflammatory processes and oxidative stress in the surrounding tissues (41).

PVAT is crucial in the intricate two-way communication with the vascular wall (Table 2). Through this interaction, PVAT can react to paracrine signals released by the vascular cells, resulting in phenotypic alterations in the adipocytes located within the PVAT. As a result of these alterations, the composition of the secretory products released by PVAT is modified. These secretory products subsequently exert paracrine effects on the vascular wall itself, influencing its function and behavior (18, 60). It plays a crucial role in maintaining vascular health by secreting a variety of vasoactive factors. These factors encompass a diverse range of substances, including (61–63). Through the release of these biologically active molecules, PVAT contributes to the modulation of vascular functions, which can influence processes such as blood flow and vascular tone (18, 64). Under conditions of heightened oxidative stress in the vascular system, substances resulting from lipid peroxidation, such as 4-hydroxynonenal (4-HNE), are emitted by the walls of blood vessels (65). The substances infiltrate the adjacent PVAT, subsequently activating signaling pathways associated with peroxisome proliferator-activated receptor (PPAR)-γ in the perivascular adipocytes. This activation leads to elevated levels of adiponectin expression and release from PVAT, an essential factor for diminishing oxidative stress in blood vessels, acting as a protective mechanism against oxidative damage in the vascular environment (66, 67).

**Table 2:** Main bioactive components in PVAT: biological functions in the vascular bed.

Main bioactive components	Specific substances	Function	Physiological role	Pathological association
Adipokines	Adiponectin	Anti—inflammatory, enhance insulin sensitivity, anti—athero (42)	Regulate metabolism, reduce inflammation, maintain vascular health (43)	Linked to obesity, type 2 diabetes mellitus (T2DM), CVD; low in obese (44)
	SFRP5	Anti—inflammatory, regulate fat tissue inflammation	Inhibit inflammation pathways in fat	Related to obesity—related issues, useful in anti—inflammation (45)
	Omentin	Anti—inflammatory, improve insulin sensitivity.	Regulate inflammation, glucose uptake, and metabolism	Changed in obesity/diabetes, a biomarker and target (46)
	Apelin	Regulate cardio, angiogen, water—salt, partly anti—inflammatory	Affect heart, blood vessels, fluid balance	Linked to cardio diseases, obesity, metabolic syndrome (47)
	Leptin	Regulate appetite/energy, anti—inflammatory	Control weight, balance fat/energy, reproductive/immune role	Obese have leptin resistance, related to obesity/eating (48)
	Chemerin	Regulate fat cell, immune cell, inflammation	Guide immune cells, affect fat cells in immunity/metabolism	Linked to obesity, inflammation; levels up in obese (49)
	Visfatin	Insulin—like, regulate blood sugar, inflammation	Promote glucose uptake, regulate inflammation	Related to obesity, insulin resistance, diabetes (50, 51)
	Resistin	Maybe involved in insulin resistance, pro—inflammatory	Affect insulin, blood sugar, and inflammation	Linked to obesity and T2DM risk (52)
	Wnt5a	In fat cell metabolism/differentiation, may regulate inflammation	Affect fat cell features, communication, and signaling	Linked to fat metabolism issues like obesity (53)
	RBP4	Transport retinol, related to insulin resistance, pro—inflammatory	Affect fat/liver metabolism, insulin signaling	Linked to obesity, T2DM, metabolic syndrome; risk factor (54)
Cytokines	IL-6 (adipose)	Pro—inflammatory	Mediate adipose tissue response	Elevated in obesity, related to CVD (55)
	TNF-α (adipose)	Pro—inflammatory	Induce adipose inflammation	Linked to insulin resistance, CVD (56)
Gas Messengers	NO	Relax vessels, regulate tone, immune	Relax PVAT—related vessels	Decrease in PVAT issues leads to problems (57)
	H₂S	Relax vessels, anti—oxidant, anti—inflammatory (58)	Protect PVAT, regulate tension	Abnormal levels cause PVAT dysfunction (59)

The balance between adipokines may be crucial in determining the overall impact of PVAT on cardiovascular health (Figure 1B). Understanding the complex interactions among these factors could yield new insights into the pathophysiology of obesity-related CVD and inform the development of novel treatment strategies. Adiponectin, a vasodilatory adipokine primarily secreted by adipocytes, plays an important role in the anti-contractile function of PVAT and is significant for regulating insulin sensitivity and glucose metabolism (68). In instances of obesity, the levels of plasma adiponectin decrease, which aids in the progression of hypertension (69), atherosclerosis (70), and diabetes (71). Leptin is another prominent adipokine that plays a crucial role in regulating appetite and body weight (72). In instances of obesity, there is an increase in leptin secretion; nonetheless, it is common for individuals to develop resistance to leptin (73). Leptin is capable of promoting vasodilation via mechanisms that rely on the endothelium as well as those that do not. In contrast, consistently elevated leptin levels may contribute to vasoconstriction (74, 75). Additionally, high levels of leptin have been associated with obesity-related conditions, including myocardial infarction (76, 77) and stroke (78). Apelin expression increases in obesity and interacts with the abnormal function of PVAT during the onset and progression of CVD, such as hypertension (79) and atherosclerosis (80). Visfatin is highly secreted by VAT and PVAT, and it appears to play a role in atherosclerosis while being associated with a vasopressor effect (81). Omentin is an adipokine that possesses both anti-contractile and anti-inflammatory characteristics. It enhances the bioavailability of NO, exhibits antioxidant properties, and provides protective effects against ischemic stroke by improving atherosclerosis (82, 83).

In addition to adipokines, PVAT also secretes various other factors, including cytokines, growth factors, and gaseous messengers. These factors can influence vascular function and play a role in the pathophysiology of CVDs. For instance, interleukin-6 (IL-6) (84) and tumor necrosis factor-alpha (TNF-α) (85) are pro-inflammatory cytokines released from PVAT in conditions such as obesity. Vascular inflammation, oxidative stress, and dysfunction of the endothelium can be initiated by these cytokines, which, in turn, play a role in the progression of CVDs (86). Additionally, hydrogen sulfide (H₂S), a gaseous messenger released from PVAT, exhibits antioxidant and vasodilatory effects under physiological conditions (87). However, in obesity and other pathological states, the production of H₂S may be altered, resulting in modifications to vascular function (88, 89). NO is a well-recognized vasodilator that plays a significant role in mediating the anti-contractile effect in PVAT (90). Initially, obesity may induce an adaptive increase in NO levels; however, chronic obesity ultimately results in diminished NO bioavailability (91). NO-dependent endothelial dysfunction is a critical initial step in the development of CVDs, particularly in the pathogenesis of atherosclerosis and hypertension (92). Furthermore, NO dysfunction also impacts myocardial remodeling (93). The above conclusions are mainly based on the results of a large number of animal experiments.

Recent studies reveal that PVAT transfers functional mitochondria to endothelial cells via extracellular vesicles (EVs), primarily exosomes, thereby enhancing vascular bioenergetics and redox balance (94–96). This intercellular mitochondrial transfer is mediated through exosomal packaging mechanisms regulated by the Endosomal Sorting Complex Required for Transport (ESCRT) and Rab GTPase proteins, which facilitate the formation of multivesicular bodies (MVBs) and subsequent vesicle docking to recipient cells (97). Exosomes selectively encapsulate mitochondria via “mitochondrial sorting” signals involving syntaxin-17 and HSP90, ensuring functional organelle delivery (98). However, the depot-specific efficiency, with thoracic PVAT exhibiting greater efficacy than abdominal PVAT, may arise from higher exosomal yield and enhanced mitochondrial quality control (e.g., PINK1/Parkin-mediated mitophagy) in thoracic depots (31). Notably, obesity disrupts this process by inducing mitochondrial dysfunction (e.g., reduced oxidative phosphorylation and mtDNA mutations) in transferred organelles, while concurrently suppressing protective adipokines such as Neuregulin-4 (Nrg4) (99). Nrg4, secreted by adipocytes, attenuates metabolic inflammation by repressing pro-inflammatory macrophage polarization and restoring T cell function, thereby preserving PVAT homeostasis and exosomal mitochondrial integrity (100). These findings challenge the “Paracrine-Centric View” associated with PVAT and highlight Nrg4 agonism as a potential strategy to counteract obesity-induced mitochondrial transfer deficits.

### 2.3 Immune and inflammatory changes of PVAT in pathological states

#### 2.3.1 Obesity-induced PVAT remodeling and immune dysregulation

Obesity profoundly alters the phenotype of PVAT, characterized by white adipocyte hypertrophy and hyperplasia within this depot (101–103). Excess body weight may also promote the conversion of aortic BAT into a WAT-like phenotype (104). These adipocyte changes are metabolically significant, as hypertrophy is linked to increased inflammation and insulin resistance (105). Critically, obesity drives remarkable shifts in the immune cell landscape of PVAT. Populations of macrophages, neutrophils, dendritic cells, eosinophils, natural killer cells, B cells, and T cells exhibit altered abundance and function (2). Macrophages, the most extensively studied immune component, increase in number and activation state (polarization) during obesity, with a distinct shift towards pro-inflammatory M1 over anti-inflammatory M2 polarization (106). This M1 dominance is associated with insulin resistance and hypertension (103). Additional immune cells also play a role in the inflammatory response within PVAT, though they do so via different mechanisms. For example, Neutrophils contribute to early macrophage polarization through the release of CCL2 and TNF in response to high-fat diets (107). Dendritic cells are found in minimal quantities within the lean phenotype but are elevated in specific adipose regions when exposed to a high-fat diet (107, 108). Additionally, mast cells in adipose tissue demonstrate an increase during obesity, although not as prominently as macrophages (109, 110). In contrast, eosinophils show a decline in obesity, and their emerging function as potential key regulators of metabolic stability is significant (111, 112). The combined populations of B (113, 114) and T cells (115, 116) make up the second largest group of immune cells located in adipose tissue, following the macrophages found within this tissue. In the context of obesity, there is a greater overall presence of T cells, with their expression varying by depot (117). This dysfunctional state of PVAT contributes to increased vascular tone, vessel stiffness, activation of the renin-angiotensin-aldosterone system (RAAS), and diminished anti-contractile effects, promoting peripheral resistance and hypertension (118, 119).

#### 2.3.2 Reciprocal interactions between diabetes and PVAT

A complex bidirectional relationship exists between diabetes and PVAT dysfunction. Hyperglycemia induces systemic oxidative stress and the overproduction of ROS (120, 121), which activate inflammatory pathways such as NF-κB. This activation triggers the infiltration of inflammatory cells into PVAT and the release of cytokines, including IL-6 and TNF-α (121, 122), establishing a chronic inflammatory state. Concurrently, dysfunction in PVAT adipocytes manifests as increased lipolysis and the release of free fatty acids, which induce lipotoxicity (123). While also reducing the secretion of the beneficial adipokine adiponectin. This deficiency in adiponectin impairs vascular function by decreasing NO bioavailability and increasing endothelin-1, thereby disrupting the balance of vascular tone (124). Conversely, dysfunctional PVAT actively contributes to the pathogenesis of diabetes. Disrupted adipokine secretion (e.g., decreased adiponectin and elevated levels of leptin, resistin, retinol binding protein 4, TNF-α, and IL-6) impairs insulin sensitivity in peripheral tissues. Inflammatory factors such as TNF-α and IL-6 interfere with insulin signaling by activating serine kinases (e.g., via JNK, NF-κB, and JAK-STAT pathways) (Figure 1C), leading to the phosphorylation of insulin receptor substrates and the blockade of signal transduction (125–127). Furthermore, vascular lesions driven by PVAT can compromise tissue perfusion, hindering the systemic delivery of insulin and glucose.

#### 2.3.3 The role of PVAT in atherosclerosis development

PVAT has a pivotal role in the development of atherosclerosis, a condition characterized by the thickening and hardening of arterial walls. This tissue functions by secreting various adipokines and inflammatory mediators, including IL-6 and TNF-α. These substances activate inflammatory signaling pathways within both vascular endothelial cells and vascular smooth muscle cells (128). For instance, upon stimulation by tumor necrosis factor-α, endothelial cells activate the nuclear factor-κB signaling pathway, leading to increased expression of intercellular adhesion molecule-1 and vascular cell adhesion molecule-1. This enhances the adhesion of inflammatory cells to the endothelial surface of blood vessels (129). Concurrently, inflammatory mediators induce vascular endothelial cells to secrete chemokines, including monocyte chemoattractant protein-1. Guided by the gradient of these chemokines, monocytes and other inflammatory cells traverse the gaps between endothelial cells and migrate into the subintima (130, 131). Additionally, PVAT serves as a significant source of ROS. The substantial production of ROS can oxidatively modify low-density lipoprotein, resulting in the formation of oxidized low-density lipoprotein, which impairs the relaxation function of endothelial cells (132). Ultimately resulting in thickening of the blood vessel wall and narrowing of the lumen, thereby effectively promoting the development of atherosclerosis (Figure 1D).

#### 2.3.4 Relationship between hypertension and PVAT function

Hypertension exerts significant structural and functional impacts on PVAT. Chronically elevated pressure stimulates the differentiation of precursor cells into adipocytes and promotes lipid accumulation in existing adipocytes (133). Additionally, hypertension remodels the PVAT extracellular matrix by increasing collagen and fibronectin deposition, which disrupts local substance exchange and signaling. Additionally, hypertension alters the extracellular matrix components of PVAT, leading to increased deposition of collagen and fibronectin. These structural modifications disrupt the exchange of substances and signal transduction within PVAT, thereby impairing its normal physiological function (134, 135). Functionally, the hemodynamic alterations and heightened oxidative stress induced by hypertension contribute to PVAT dysfunction. Oxidative stress generates substantial amounts of ROS, which can damage cellular components in PVAT. Furthermore, hypertension disrupts the balance of vasoactive substances and inflammatory factors secreted by PVAT, transitioning it from a normal physiological regulatory state to a pathological state that promotes vasoconstriction and inflammation, further aggravating hypertension (136, 137). Reciprocally, PVAT critically influences blood pressure regulation and becomes a key site for immune cell infiltration in hypertension (138). Infiltrating immune cells, such as T cells, release effector cytokines—including interferon-gamma (IFN-γ), interleukin-17 (IL-17), TNF-α, and IL-6 which initiate biological cascades that contribute to vascular dysfunction, heightened oxidative stress, and increased vascular stiffness. These PVAT-driven inflammatory and vascular responses significantly contribute to the pathogenesis of hypertension and its complications (139, 140) (Figure 1E).

## 3 PVAT as a source of biomarkers

### 3.1 Advancements in imaging techniques for assessing PVAT inflammation

Computed Tomography (CT) imaging has established itself as the benchmark for effectively visualizing and characterizing PVAT (141). This is largely attributed to its exceptional spatial resolution, which allows for detailed imaging of structures within the body. Moreover, the distinct attenuation signals generated by adipose tissue enhance the accuracy and clarity of the images obtained. As a result, CT Imaging proves to be an invaluable tool in the study and analysis of PVAT, facilitating better understanding and insights into its role in various physiological and pathological conditions (142). The fat attenuation index (FAI) serves as a measure aimed at quantifying the three-dimensional attenuation gradients identified in coronary computed tomography angiography (CCTA) imaging of PVAT (141). FAI has received both biological and clinical validation, and it is now acknowledged as the main imaging biomarker for coronary inflammation sourced from CCTA. Standardized FAI evaluations are capable of accounting for side branches, thus yielding a more precise evaluation of PVAT inflammation (143, 144). In addition, the artificial intelligence (AI)-driven imaging biomarker (FRP) significantly improves the prediction of cardiac risk (143).

However, various imaging modalities, including magnetic resonance imaging (MRI) (145), positron emission tomography (PET), and ultrasound, offer promising applications for evaluating PVAT. MRI allows for the assessment of PVAT volume around major arteries, including the aorta, and has demonstrated an independent association with markers of subclinical atherosclerosis (146). PET provides functional insights into the metabolic activity of adipose tissue; however, its low spatial resolution limits its effectiveness in evaluating PVAT around coronary arteries (142). Although ultrasound may yield some surrogate measurements of EAT (147–149), its limited ability to distinguish adipose tissue from other structures, combined with its operator-dependent nature, reduces its utility in assessing PVAT in the coronary region.

### 3.2 Circulating biomarkers in evaluating PVAT function

In addition to imaging biomarkers, circulating biomarkers, including adipokines and cytokines, have potential applications in evaluating PVAT function and predicting CVDs (150). For instance, plasma adiponectin levels are diminished in individuals with obesity and diabetes, and lower adiponectin levels have been linked to an elevated risk of CVDs (151). Similarly, plasma levels of IL-6 and TNF-α are increased in obesity and other pathological conditions, with high levels of these cytokines associated with a greater risk of CVDs (152). However, it is important to note that circulating biomarkers are not specific to PVAT function and may be influenced by other factors, such as systemic inflammation and tissue damage.

## 4 PVAT as a therapeutic target

### 4.1 Lifestyle modifications for enhancing PVAT function and reducing cardiovascular risk

Lifestyle modifications, including diet and exercise, hold significant promise for enhancing PVAT function and mitigating the risk of CVDs (153–155). In humans, exercise-induced weight loss is more effective than dieting alone in improving circulating adipokine profiles and insulin resistance (156, 157). High-intensity interval training has been particularly effective in reducing total adipose tissue and visceral adipose tissue (VAT) mass in adults (158, 159), as well as enhancing cardiorespiratory fitness in obese children (158, 160).

### 4.2 The role of novel pharmacological strategies in restoring PVAT function in CVD

PVAT has a well-established significance in the development and progression of vascular diseases, making it a promising target for innovative therapeutic strategies in cardiovascular medicine. Research has demonstrated that current anti-diabetic medications can partly mediate their positive cardiovascular effects through their interactions with adipose tissues, including PVAT (161). For example, glucagon-like peptide-1 (GLP-1) receptor agonists facilitate adipocyte differentiation and enhance overall adipocyte health (162, 163). GLP-1 and its agonists exert various direct effects on PVAT biology, including the reduction of lipid accumulation (164) and the promotion of lipogenesis (165). Furthermore, GLP-1 receptor agonists upregulate the expression of adipokines (166) and promote M2 macrophage polarization (167), clinical studies have demonstrated that administering the GLP-1 analogue liraglutide to individuals with obesity results in favorable modifications in plasma lipid profiles and a reduction in apolipoprotein B levels, which could significantly lower the risk of CVDs (168–171). Additionally, sodium-glucose cotransporter 2 inhibitors (SGLT2-i) have been found to exert beneficial effects on human PVAT, improving mitochondrial efficiency, reducing oxidative stress and inflammation, and enhancing tissue function (172). The SGLT2 inhibitor empagliflozin is notable for its ability to enhance the browning of WAT and activate resident M2 macrophages, which helps decrease inflammation and insulin resistance linked to obesity, as demonstrated in animal research (173, 174). In human subjects, treatment with empagliflozin is linked to a decrease in the volume of EAT, which is critically associated with cardiometabolic risks (175, 176). Moreover, statins are known to decrease vascular inflammation, and their administration correlates with significant changes in the phenotype of PVAT following instances of acute coronary syndrome (ACS) and the commencement of statin treatment (177, 178). Other potential methods for restoring the health of PVAT include the use of PPAR-γ agonists such as rosiglitazone (179, 180). It is essential to acknowledge the recognized negative impacts on individuals suffering from congestive heart failure. Furthermore, agonists of the cannabinoid CB1 receptor might facilitate the release of H_2_S from PVAT (181, 182). Mineralocorticoid receptor blockers have been shown to reduce adipose tissue inflammation and increase adiponectin levels (183, 184). However, the specific effects of current drugs on the PVAT remain unquantified, which poses a risk of off-target consequences. Therefore, it is essential to examine this issue from a more neutral perspective and to conduct further research.

### 4.3 Effect of surgical intervention on PVAT function

In patients with type 2 diabetes and obesity, undergoing metabolic surgery (defined as procedures that influence metabolism by inducing weight loss and altering gastrointestinal physiology) has been associated with a significantly reduced risk of major adverse cardiovascular events (MACE) compared to traditional nonsurgical management approaches (185). This finding highlights the potential of metabolic surgical interventions to enhance cardiovascular outcomes for this patient population, underscoring the necessity for a shift in treatment strategies. The deterioration of the anticontractile properties of PVAT observed in obesity is partly attributable to a reduction in adiponectin levels within the PVAT. This hormone is crucial for maintaining vascular health. Importantly, the weight loss achieved through bariatric surgery has the potential to restore adiponectin levels, which may lead to improvements in PVAT function and, consequently, cardiovascular health (186). In contrast, procedures such as abdominal liposuction have not shown significant benefits in correcting the metabolic abnormalities associated with obesity, thereby highlighting the limitations of these methods in addressing the complex nature of obesity-related health issues (187).

### 4.4 Targeting gut microbiota as a therapeutic approach to enhance PVAT function and vascular health

The gut microbiota plays a substantial role in influencing PVAT through various mechanisms, particularly by producing metabolites and regulating immune responses (188). Among these metabolites, short-chain fatty acids produced by gut microbiota have been found to enhance the metabolic activity of adipocytes within the PVAT, which can lead to a reduction in inflammation levels. On the other hand, the presence of trimethylamine-N-oxide can trigger oxidative stress and inflammation in PVAT, negatively affecting its function. Additionally, emerging research in the field of cancer therapy shows that gut microbiota can regulate immune responses and produce metabolites that impact cancer development and treatment responses, suggesting its role in PVAT may have implications for broader health aspects related to cancer and vascular health (189, 190). Maintaining a healthy balance of gut microbiota is crucial, as it regulates immune cells that are essential for preserving PVAT homeostasis. When dysbiosis occurs, this balance is disrupted, potentially compromising both the structure and the functional integrity of PVAT. This finding highlights the promising potential of targeting gut microbiota as a therapeutic approach to enhance PVAT function and overall vascular health (191).

### 4.5 Other potential therapeutic targets

The activation of β3-adrenergic receptors (β3-AR) by mirabegron presents another fascinating avenue for bolstering vascular health. Research has indicated that this pharmacological agent can prevent serious vascular conditions such as aortic dissection and aneurysm by promoting lymphangiogenesis within PVAT (192). Furthermore, colchicine has shown promise in inhibiting inflammatory responses in PVAT, as well as curbing abnormal cellular behaviors such as proliferation and migration that can lead to vascular complications (193–195). The role of perivascular relaxing factors, including methyl palmitate, is equally critical in managing PVAT and enhancing vascular functionality. These factors facilitate several important processes, including the relaxation of vascular smooth muscle, reduction of vascular resistance, and improvement of blood perfusion. Additionally, they work to inhibit inflammatory responses and decrease both inflammatory cell infiltration and mediator production (196, 197). Collectively, these multifaceted effects play a vital role in addressing CVDs associated with PVAT dysfunction. Despite the promising implications of these interventions, it is essential to conduct further research to ascertain their specific effects and ensure their safety for clinical applications.

## 5 Discussion and outlook

### 5.1 Discussion

The evolving recognition of PVAT as a pivotal regulator of vascular homeostasis and a driver of CVD underscores its potential as a therapeutic target. However, translating mechanistic insights into clinical applications remains fraught with challenges, necessitating a critical reevaluation of current paradigms and the integration of cutting-edge methodologies. While recent advancements in single-cell omics, imaging biomarkers, and therapeutic strategies have illuminated PVAT's spatiotemporal complexity, significant gaps still persist in reconciling its depot-specific heterogeneity with systemic vascular pathology. Although animal experiments have shown that β3-AR, methyl palmitate, and cannabinoid CB1 receptor agonists can alleviate the inflammatory response of PVAT (181, 182, 192), it is crucial to note that due to differences in PVAT composition and function across species, the applicability of preclinical findings (primarily from rodent models) to human pathophysiology requires rigorous validation. Furthermore, gender and age profoundly influence PVAT biology and CVD risk, though mechanistic insights into these effects remain limited (198, 199). For instance, postmenopausal women exhibit accelerated PVAT inflammation linked to estrogen loss (200, 201), and aging-associated PVAT fibrosis exacerbates endothelial dysfunction (122, 202).

To address these complexities, in this review, we systematically synthesized the molecular crosstalk, spatial heterogeneity, and therapeutic potential of PVAT in the pathogenesis of CVD. Concurrently, we highlighted three limitations of previous studies: (1) the oversimplified assumption of PVAT homogeneity, (2) the paracrine-centric view that overshadows mechanotransduction pathways, and (3) the unquantified depot-specific effects of existing therapies.

Given this landscape, the emerging role of PVAT in cardiovascular pathophysiology necessitates a paradigm shift from observational research to precision therapeutics. While single-cell omics elucidate its cellular heterogeneity—such as UCP1 + thermogenic, ADIPOQ + endocrine, and COL1A1 + fibro-inflammatory adipocytes—and depot-specific metabolic programming, clinical translation is hindered by insufficient human models and an overreliance on rodent data. Although advanced imaging biomarkers, such as fat attenuation index (FAI), and AI-driven radiomics enhance the detection of coronary inflammation (141, 142); standardization gaps combined with systemic confounding factors limit their practical utility. Consequently, therapeutic innovation should prioritize PVAT-centric strategies, including macrophage-targeted PPAR-γ delivery and CRISPR-mediated ADIPOQ activation. Despite the preclinical promise shown by agents like β3-adrenergic receptor agonists (192) and colchicine (194), human trials remain limited. Hence, global consortia are essential to standardize protocols, address disparities, and ethically integrate AI-driven platforms. Ultimately, through bridging mechanistic depth with clinical pragmatism, PVAT research has the potential to redefine precision cardiology and mitigate residual cardiovascular risk.

### 5.2 Future research directions

**Figure 2:**
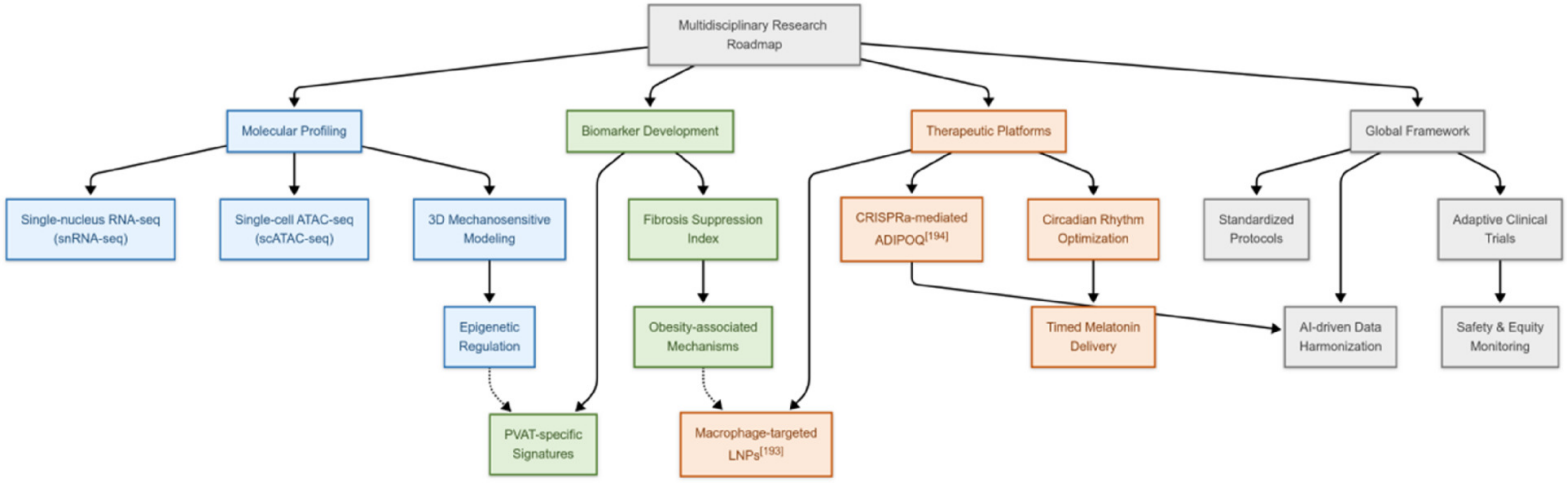
Multidisciplinary research roadmap for PVAT.

To address the specific gaps and broader translational challenges, such as species differences, inadequacies in human models, biomarker standardization, and the necessity for human trials, we propose a multidisciplinary roadmap that integrates molecular insights with translational applications. Firstly, single-cell and spatial multi-omics techniques (e.g., snRNA-seq and single-cell ATAC sequencing) will elucidate PVAT's cellular diversity and epigenetic mechanisms, complemented by dynamic three-dimensional models to validate mechanosensitive pathways. Additionally, the development of clinical biomarkers requires cross-population validation of PVAT-specific signatures, while also addressing obesity-related fibrotic suppression. Moreover, therapeutic innovation depends on nanotechnology-enabled delivery systems [e.g., macrophage-targeted lipid nanoparticles (203)] and CRISPR activation-mediated ADIPOQ activation (204), combined with circadian optimization (e.g., timed melatonin administration). Finally, global consortia must standardize protocols and utilize AI-driven data harmonization to accelerate discovery, while adaptive trials address sex and racial disparities and ensure gene-editing safety (Figure 2).

Moving forward, precision therapies will concentrate on cell-specific PVAT targets by employing systems biology and network pharmacology. Personalized lifestyle modifications and surgical interventions will be refined based on long-term outcome data, while mechanistic studies of gut microbiota and PVAT will inform targeted interventions. By emphasizing mechano-metabolic pathways, microbiota interactions, and ethical considerations, this roadmap seeks to transform PVAT biology into stratified cardiovascular therapies, thereby reducing residual risk and enhancing disease management through molecular precision.

## 6 Conclusion

PVAT serves as a critical regulator of vascular homeostasis, with its dysfunction contributing to cardiovascular pathology through paracrine imbalance, metabolic dysregulation, and extracellular matrix remodeling. Advances in imaging techniques and circulating biomarkers now facilitate the early detection of PVAT abnormalities, while emerging therapies, such as SGLT2 inhibitors and circadian rhythm modulation, show promise in restoring PVAT function. However, challenges persist in standardizing diagnostic protocols, optimizing tissue-specific drug delivery, and evaluating long-term safety. Future research ought to prioritize translational studies and multidisciplinary collaboration to integrate PVAT-targeted strategies into precision cardiovascular medicine, ultimately aiming to reduce residual disease risks and enhance clinical outcomes.

## References

[1] Antoniades C Tousoulis D Vavlukis M Fleming I Duncker DJ Eringa E Perivascular adipose tissue as a source of therapeutic targets and clinical biomarkers. Eur Heart J. (2023) 44(38):3827–44. 10.1093/eurheartj/ehad484 37599464 PMC10568001

[2] Saxton SN Clark BJ Withers SB Eringa EC Heagerty AM. Mechanistic links between obesity, diabetes, and blood pressure: role of perivascular adipose tissue. Physiol Rev. (2019) 99(4):1701–63. 10.1152/physrev.00034.2018 31339053

[3] Meijer RI Hoevenaars FPM Serné EH Yudkin JS Kokhuis TJA Weijers EM JNK2 in myeloid cells impairs insulin’s vasodilator effects in muscle during early obesity development through perivascular adipose tissue dysfunction. Am J Physiol Heart Circ Physiol. (2019) 317(2):H364–74. 10.1152/ajpheart.00663.2018 31149833

[4] Chen JY Wu YP Li CY Jheng HF Kao LZ Yang CC PPARγ activation improves the microenvironment of perivascular adipose tissue and attenuates aortic stiffening in obesity. J Biomed Sci. (2021) 28(1):22. 10.1186/s12929-021-00720-y 33781257 PMC8008548

[5] Wang Y Wang X Chen Y Zhang Y Zhen X Tao S Perivascular fat tissue and vascular aging: a sword and a shield. Pharmacol Res. (2024) 203:107140. 10.1016/j.phrs.2024.107140 38513826

[6] Azul L Leandro A Boroumand P Klip A Seiça R Sena CM. Increased inflammation, oxidative stress and a reduction in antioxidant defense enzymes in perivascular adipose tissue contribute to vascular dysfunction in type 2 diabetes. Free Radic Biol Med. (2020) 146:264–74. 10.1016/j.freeradbiomed.2019.11.002 31698080

[7] Stanek A Brożyna-Tkaczyk K Myśliński W. The role of obesity-induced perivascular adipose tissue (PVAT) dysfunction in vascular homeostasis. Nutrients. (2021) 13(11):3843. 10.3390/nu13113843 34836100 PMC8621306

[8] Rafeh R Viveiros A Oudit GY El-Yazbi AF. Targeting perivascular and epicardial adipose tissue inflammation: therapeutic opportunities for cardiovascular disease. Clin Sci (Lond). (2020) 134(7):827–51. 10.1042/CS20190227 32271386

[9] Liu C-L Ren J Wang Y Zhang X Sukhova GK Liao M Adipocytes promote interleukin-18 binding to its receptors during abdominal aortic aneurysm formation in mice. Eur Heart J. (2020) 41(26):2456–68. 10.1093/eurheartj/ehz856 31821481 PMC8453281

[10] Chang L Villacorta L Li R Hamblin M Xu W Dou C Loss of perivascular adipose tissue on peroxisome proliferator-activated receptor-γ deletion in smooth muscle cells impairs intravascular thermoregulation and enhances atherosclerosis. Circulation. (2012) 126(9):1067–78. 10.1161/CIRCULATIONAHA.112.104489 22855570 PMC3493564

[11] Chang L Xiong W Zhao X Fan Y Guo Y Garcia-Barrio M Bmal1 in perivascular adipose tissue regulates resting-phase blood pressure through transcriptional regulation of angiotensinogen. Circulation. (2018) 138(1):67–79. 10.1161/CIRCULATIONAHA.117.029972 29371216 PMC6030431

[12] Dorothea S-A Häring HU. Perivascular adipose tissue: an unique fat compartment relevant for the cardiometabolic syndrome. Rev Endocr Metab Disord. (2016) 17(1):51–60. 10.1007/s11154-016-9346-3 26995737

[13] Gu P Hui X Zheng Q Gao Y Jin L Jiang W Mitochondrial uncoupling protein 1 antagonizes atherosclerosis by blocking NLRP3 inflammasome-dependent interleukin-1β production. Sci Adv. (2021) 7(50):eabl4024. 10.1126/sciadv.abl4024 34878840 PMC8654294

[14] Padilla J Jenkins NT Vieira-Potter VJ Laughlin MH. Divergent phenotype of rat thoracic and abdominal perivascular adipose tissues. Am J Physiol Regul Integr Comp Physiol. (2013) 304(7):R543–52. 10.1152/ajpregu.00567.2012 23389108 PMC3627942

[15] Mestres-Arenas A Villarroya J Giralt M Villarroya F Peyrou M. A differential pattern of batokine expression in perivascular adipose tissue depots from mice. Front Physiol. (2021) 12:714530. 10.3389/fphys.2021.714530 34421656 PMC8373243

[16] Iacobellis G. Epicardial adipose tissue in contemporary cardiology. Nat Rev Cardiol. (2022) 19(9):593–606. 10.1038/s41569-022-00679-9 35296869 PMC8926097

[17] Iacobellis G Bianco AC. Epicardial adipose tissue: emerging physiological, pathophysiological and clinical features. Trends Endocrinol Metab. (2011) 22(11):450–7. 10.1016/j.tem.2011.07.003 21852149 PMC4978122

[18] Ahmadieh S Kim HW Weintraub NL. Potential role of perivascular adipose tissue in modulating atherosclerosis. Clin Sci (Lond). (2020) 134(1):3–13. 10.1042/CS20190577 31898749 PMC6944729

[19] Cinti S. Adipocyte differentiation and transdifferentiation: plasticity of the adipose organ. J Endocrinol Invest. (2002) 25(10):823–35. 10.1007/BF03344046 12508945

[20] Himms-Hagen J Melnyk A Zingaretti MC Ceresi E Barbatelli G Cinti S. Multilocular fat cells in WAT of CL-316243-treated rats derive directly from white adipocytes. Am J Physiol Cell Physiol. (2000) 279(3):C670–81. 10.1152/ajpcell.2000.279.3.C670 10942717

[21] Rahbani JF Bunk J Lagarde D Samborska B Roesler A Xiao H Parallel control of cold-triggered adipocyte thermogenesis by UCP1 and CKB. Cell Metab. (2024) 36(3):526–40.e7. 10.1016/j.cmet.2024.01.001 38272036

[22] Giroud M Jodeleit H Prentice KJ Bartelt A. Adipocyte function and the development of cardiometabolic disease. J Physiol. (2022) 600(5):1189–208. 10.1113/JP281979 34555180

[23] Xiao L De Jesus DF Ju CW Wei JB Hu J Distefano-Forti A M(6)A mRNA methylation in brown fat regulates systemic insulin sensitivity via an inter-organ prostaglandin signaling axis independent of UCP1. Cell Metab. (2024) 36(10):2207–27.e9. 10.1016/j.cmet.2024.08.006 39255799 PMC11891809

[24] Angueira AR Sakers AP Holman CD Cheng L Arbocco MN Shamsi F Defining the lineage of thermogenic perivascular adipose tissue. Nat Metab. (2021) 3(4):469–84. 10.1038/s42255-021-00380-0 33846639 PMC8136151

[25] van Marken Lichtenbelt WD Vanhommerig JW Smulders NM Drossaerts JM Kemerink GJ Bouvy ND Cold-activated brown adipose tissue in healthy men. N Engl J Med. (2009) 360(15):1500–8. 10.1056/NEJMoa0808718 19357405

[26] Ye M Ruan CC Fu M Xu L Chen D Zhu M Developmental and functional characteristics of the thoracic aorta perivascular adipocyte. Cell Mol Life Sci. (2018) 76(4):777–89. 10.1007/s00018-018-2970-1 30448891 PMC11105183

[27] Long JZ Svensson KJ Tsai L Zeng X Roh HC Kong X A smooth muscle-like origin for beige adipocytes. Cell Metab. (2014) 19(5):810–20. 10.1016/j.cmet.2014.03.025 24709624 PMC4052772

[28] Vijay J Gauthier M-F Biswell RL Louiselle DA Johnston JJ Cheung WA Single-cell analysis of human adipose tissue identifies depot and disease specific cell types. Nat Metab. (2020) 2(1):97–109. 10.1038/s42255-019-0152-6 32066997 PMC7025882

[29] Li X Ma Z Zhu YZ. Regional heterogeneity of perivascular adipose tissue: morphology, origin, and secretome. Front Pharmacol. (2021) 12:697720. 10.3389/fphar.2021.697720 34239444 PMC8259882

[30] Wang Z Lu H Garcia-Barrio M Guo Y Zhang J Chen YE RNA sequencing reveals perivascular adipose tissue plasticity in response to angiotensin II. Pharmacol Res. (2022) 178:106183. 10.1016/j.phrs.2022.106183 35306139

[31] Hwej A Al-Ferjani A Alshuweishi Y Naji A Kennedy S Salt IP. Lack of AMP-activated protein kinase-α1 reduces nitric oxide synthesis in thoracic aorta perivascular adipose tissue. Vasc Pharmacol. (2024) 157:107437. 10.1016/j.vph.2024.107437 39433170

[32] Wilcox CS Herbert C Wang C Ma Y Sun P Li T Signals from inflamed perivascular adipose tissue contribute to small-vessel dysfunction in women with the human immunodeficiency virus. J Infect Dis. (2024) 230(1):67–77. 10.1093/infdis/jiae094 39052698 PMC11272057

[33] Krawczyńska A Herman AP Antushevich H Bochenek J Wojtulewicz K Zięba DA. The influence of photoperiod on the action of exogenous leptin on gene expression of proinflammatory cytokines and their receptors in the thoracic perivascular adipose tissue (PVAT) in ewes. Mediators Inflamm. (2019) 2019:7129476. 10.1155/2019/7129476 31780867 PMC6875191

[34] Zhang ZB Ruan CC Lin JR Xu L Chen XH Du YN Perivascular adipose tissue-derived PDGF-D contributes to aortic aneurysm formation during obesity. Diabetes. (2018) 67(8):1549–60. 10.2337/db18-0098 29794241

[35] Jüttner AA Ataei Ataabadi E Golshiri K de Vries R Garrelds IM Danser AHJ Adiponectin secretion by perivascular adipose tissue supports impaired vasodilation in a mouse model of accelerated vascular smooth muscle cell and adipose tissue aging. Vascul Pharmacol. (2024) 154:107281. 10.1016/j.vph.2024.107281 38320678

[36] Kagota S Maruyama-Fumoto K Iwata S Shimari M Koyanagi S Shiokawa Y Perivascular adipose tissue-enhanced vasodilation in metabolic syndrome rats by apelin and N -acetyl^−^l-cysteine-sensitive factor(s). Int J Mol Sci. (2018) 20(1):106. 10.3390/ijms20010106 30597883 PMC6337496

[37] Nel S Strijdom H Genis A Everson F Van Wijk R Kotzé SH. A histomorphometric study on the effects of antiretroviral therapy (ART) combined with a high-calorie diet (HCD) on aortic perivascular adipose tissue (PVAT). Acta Histochem. (2017) 119(5):555–62. 10.1016/j.acthis.2017.05.009 28606728

[38] Berti L Hartwig S Irmler M Rädle B Siegel-Axel D Beckers J Impact of fibroblast growth factor 21 on the secretome of human perivascular preadipocytes and adipocytes: a targeted proteomics approach. Arch Physiol Biochem. (2016) 122(5):281–8. 10.1080/13813455.2016.1212898 27494767

[39] Kudryavtseva O Lyngsø KS Jensen BL Dimke H. Nitric oxide, endothelium-derived hyperpolarizing factor, and smooth muscle-dependent mechanisms contribute to magnesium-dependent vascular relaxation in mouse arteries. Acta Physiol (Oxf). (2024) 240(3):e14096. 10.1111/apha.14096 38258597

[40] Ozkor MA Rahman AM Murrow JR Kavtaradze N Lin J Manatunga A Differences in vascular nitric oxide and endothelium-derived hyperpolarizing factor bioavailability in blacks and whites. Arterioscler Thromb Vasc Biol. (2014) 34(6):1320–7. 10.1161/ATVBAHA.113.303136 24675657 PMC4138537

[41] Adachi Y Ueda K Nomura S Ito K Katoh M Katagiri M Beiging of perivascular adipose tissue regulates its inflammation and vascular remodeling. Nat Commun. (2022) 13(1):5117. 10.1038/s41467-022-32658-6 36071032 PMC9452496

[42] Marek G Pannu V Shanmugham P Pancione B Mascia D Crosson S Adiponectin resistance and proinflammatory changes in the visceral adipose tissue induced by fructose consumption via ketohexokinase-dependent pathway. Diabetes. (2015) 64(2):508–18. 10.2337/db14-0411 25187370

[43] Hotta K Funahashi T Bodkin NL Ortmeyer HK Arita Y Hansen BC Circulating concentrations of the adipocyte protein adiponectin are decreased in parallel with reduced insulin sensitivity during the progression to type 2 diabetes in rhesus monkeys. Diabetes. (2001) 50(5):1126–33. 10.2337/diabetes.50.5.1126 11334417

[44] Kumada M Kihara S Sumitsuji S Kawamoto T Matsumoto S Ouchi N Association of hypoadiponectinemia with coronary artery disease in men. Arterioscler Thromb Vasc Biol. (2003) 23(1):85–9. 10.1161/01.ATV.0000048856.22331.50 12524229

[45] Wang B Pan Y Yang G Cui Z Yu W Liu H Sfrp5/Wnt5a and leptin/adiponectin levels in the serum and the periarterial adipose tissue of patients with peripheral arterial occlusive disease. Clin Biochem. (2021) 87:46–51. 10.1016/j.clinbiochem.2020.11.002 33188773

[46] Yamawaki H Kuramoto J Kameshima S Usui T Okada M Hara Y. Omentin, a novel adipocytokine inhibits TNF-induced vascular inflammation in human endothelial cells. Biochem Biophys Res Commun. (2011) 408(2):339–43. 10.1016/j.bbrc.2011.04.039 21514279

[47] Yu S Zhang Y Li MZ Xu H Wang Q Song J Chemerin and apelin are positively correlated with inflammation in obese type 2 diabetic patients. Chin Med J (Engl). (2012) 125(19):3440–4. 23044303

[48] Gálvez-Prieto B Somoza B Gil-Ortega M García-Prieto CF De Las Heras AI González MC Anticontractile effect of perivascular adipose tissue and leptin are reduced in hypertension. Front Pharmacol. (2012) 3:103. 10.3389/fphar.2012.00103 22679436 PMC3367267

[49] Stejskal D Karpisek M Hanulova Z Svestak M. Chemerin is an independent marker of the metabolic syndrome in a Caucasian population–a pilot study. Biomed Pap Med Fac Univ Palacky Olomouc Czech Repub. (2008) 152(2):217–21. 10.5507/bp.2008.033 19219210

[50] Atasoy A Çakır E Ahbab S Erdoğan Döventaş Y Koldaş M Ataoğlu E Visfatin levels in hormonally inactive adrenal adenoma and their association with metabolic parameters. Turk J Med Sci. (2018) 48(3):548–53. 10.3906/sag-1709-74 29914251

[51] Eyileten T Sonmez A Saglam M Cakir E Caglar K Oguz Y Effect of renin-angiotensin-aldosterone system (RAAS) blockade on visfatin levels in diabetic nephropathy. Nephrology (Carlton). (2010) 15(2):225–9. 10.1111/j.1440-1797.2009.01173.x 20470283

[52] Pagano C Marin O Calcagno A Schiappelli P Pilon C Milan G Increased serum resistin in adults with prader-willi syndrome is related to obesity and not to insulin resistance. J Clin Endocrinol Metab. (2005) 90(7):4335–40. 10.1210/jc.2005-0293 15870134

[53] Tong S Du Y Ji Q Dong R Cao J Wang Z Expression of Sfrp5/Wnt5a in human epicardial adipose tissue and their relationship with coronary artery disease. Life Sci. (2020) 245:117338. 10.1016/j.lfs.2020.117338 31981630

[54] Ghorbanian B Wong A Iranpour A. The effect of dietary carbohydrate restriction and aerobic exercise on retinol binding protein 4 (RBP4) and fatty acid binding protein 5 (FABP5) in middle-aged men with metabolic syndrome. Br J Nutr. (2023) 130(4):553–63. 10.1017/S0007114522003580 36373560

[55] Gruzdeva OV Dyleva YA Belik EV Sinitsky MY Stasev AN Kokov AN Relationship between epicardial and coronary adipose tissue and the expression of adiponectin, leptin, and interleukin 6 in patients with coronary artery disease. J Pers Med. (2022) 12(2):129. 10.3390/jpm12020129 35207618 PMC8877574

[56] Virdis A Colucci R Bernardini N Blandizzi C Taddei S Masi S. Microvascular endothelial dysfunction in human obesity: role of TNF-α. J Clin Endocrinol Metab. (2019) 104(2):341–8. 10.1210/jc.2018-00512 30165404

[57] Carlström M Larsen FJ Nyström T Hezel M Borniquel S Weitzberg E Dietary inorganic nitrate reverses features of metabolic syndrome in endothelial nitric oxide synthase-deficient mice. Proc Natl Acad Sci U S A. (2010) 107(41):17716–20. 10.1073/pnas.1008872107 20876122 PMC2955084

[58] Szabo C. Gaseotransmitters: new frontiers for translational science. Sci Transl Med. (2010) 2(59):59ps4. 10.1126/scitranslmed.3000721 PMC3038605 21106939

[59] Szabó C. Hydrogen sulphide and its therapeutic potential. Nat Rev Drug Discov. (2007) 6(11):917–35. 10.1038/nrd2425 17948022

[60] Szasz T Webb RC. Perivascular adipose tissue: more than just structural support. Clin Sci. (2011) 122(1):1–12. 10.1042/CS20110151 PMC3966487 21910690

[61] Hancock JT Veal D. Nitric oxide, other reactive signalling compounds, redox, and reductive stress. J Exp Bot. (2021) 72(3):819–29. 10.1093/jxb/eraa331 32687173

[62] Nakladal D Sijbesma JWA Visser LM Tietge UJF Slart R Deelman LE Perivascular adipose tissue-derived nitric oxide compensates endothelial dysfunction in aged pre-atherosclerotic apolipoprotein E-deficient rats. Vascul Pharmacol. (2021) 142:106945. 10.1016/j.vph.2021.106945 34801679

[63] Lan A Xu W Zhang H Hua X Zheng D Guo R Inhibition of ROS-activated p38MAPK pathway is involved in the protective effect of H2S against chemical hypoxia-induced inflammation in PC12 cells. Neurochem Res. (2013) 38(7):1454–66. 10.1007/s11064-013-1044-x 23624824 PMC3671109

[64] Xia N Li H. The role of perivascular adipose tissue in obesity-induced vascular dysfunction. Br J Pharmacol. (2017) 174(20):3425–42. 10.1111/bph.13650 27761903 PMC5610151

[65] Hsu CG Chávez CL Zhang C Sowden M Yan C Berk BC. The lipid peroxidation product 4-hydroxynonenal inhibits NLRP3 inflammasome activation and macrophage pyroptosis. Cell Death Differ. (2022) 29(9):1790–803. 10.1038/s41418-022-00966-5 35264781 PMC9433404

[66] Zahr T Liu L Chan M Zhou Q Cai B He Y PPARγ (peroxisome proliferator-activated receptor γ) deacetylation suppresses aging-associated atherosclerosis and hypercholesterolemia. Arterioscler Thromb Vasc Biol. (2023) 43(1):30–44. 10.1161/ATVBAHA.122.318061 36453279 PMC9917767

[67] Żółkiewicz J Stochmal A Zaremba M Rudnicka L Czuwara J. Circulating peroxisome proliferator-activated receptor *γ* is elevated in systemic sclerosis. Postepy Dermatol Alergol. (2020) 37(6):921–6. 10.5114/ada.2019.84746 33603610 PMC7874880

[68] Almabrouk TAM White AD Ugusman AB Skiba DS Katwan OJ Alganga H High fat diet attenuates the anticontractile activity of aortic PVAT via a mechanism involving AMPK and reduced adiponectin secretion. Front Physiol. (2018) 9:51. 10.3389/fphys.2018.00051 29479319 PMC5812172

[69] Li Y Zheng H Yang J Zhang B Xing X Zhang Z Association of genetic variants in leptin, leptin receptor and adiponectin with hypertension risk and circulating leptin/adiponectin changes. Gene. (2023) 853:147080. 10.1016/j.gene.2022.147080 36470480

[70] Katsiki N Mantzoros C Mikhailidis DP. Adiponectin, lipids and atherosclerosis. Curr Opin Lipidol. (2017) 28(4):347–54. 10.1097/MOL.0000000000000431 28463859

[71] Baker JF England BR Wysham KD Sauer B Joseph AM Lenert A Associations between adiponectin and the development of diabetes in rheumatoid arthritis. J Clin Endocrinol Metab. (2024) 109(10):e1839–46. 10.1210/clinem/dgae010 38189426 PMC11403312

[72] Gündüz B Okimoto DK. Methyl donor supplementation alters serum leptin levels and increases appetite but not body weight in cross-fostered male Syrian hamster offspring (mesocricetus auratus). J Anim Physiol Anim Nutr (Berl). (2022) 106(5):1130–8. 10.1111/jpn.13665 34865266

[73] Simonds SE Pryor JT Ravussin E Greenway FL Dileone R Allen AM Leptin mediates the increase in blood pressure associated with obesity. Cell. (2014) 159(6):1404–16. 10.1016/j.cell.2014.10.058 25480301 PMC4259491

[74] Rodríguez A Frühbeck G Gómez-Ambrosi J Catalán V Sáinz N Díez J The inhibitory effect of leptin on angiotensin II-induced vasoconstriction is blunted in spontaneously hypertensive rats. J Hypertens. (2006) 24(8):1589–97. 10.1097/01.hjh.0000239295.17636.6e 16877962

[75] Fortuño A Rodríguez A Gómez-Ambrosi J Muñiz P Salvador J Díez J Leptin inhibits angiotensin II-induced intracellular calcium increase and vasoconstriction in the rat aorta. Endocrinology. (2002) 143(9):3555–60. 10.1210/en.2002-220075 12193570

[76] Syed AH Lohana S Aung NH Memon MK Shaikh A Memon S Correlation of leptin with acute myocardial infarction: a case control study. Cureus. (2020) 12(12):e12190. 10.7759/cureus.12190 33489600 PMC7815288

[77] Baig M Alghalayini KW Gazzaz ZJ Atta H. Association of serum omentin-1, chemerin, and leptin with acute myocardial infarction and its risk factors. Pak J Med Sci. (2020) 36(6):1183–8. 10.12669/pjms.36.6.2372 32968377 PMC7501013

[78] Saber H Himali JJ Shoamanesh A Beiser A Pikula A Harris TB Serum leptin levels and the risk of stroke: the Framingham study. Stroke. (2015) 46(10):2881–5. 10.1161/STROKEAHA.115.009463 26337973 PMC4589501

[79] Zhang Q Shen Y Niloy SI O’rourke ST Sun C. Chronic effects of apelin on cardiovascular regulation and angiotensin II-induced hypertension. Pharmaceuticals (Basel. (2023) 16(4):600. 10.3390/ph16040600 37111357 PMC10145143

[80] Liu LQ Zhang P Qi YZ Li H Jiang YH Yang CH. Quercetin attenuates atherosclerosis via modulating apelin signaling pathway based on plasma metabolomics. Chin J Integr Med. (2023) 29(12):1121–32. 10.1007/s11655-023-3645-9 37656412

[81] Kärberg K Forbes A Lember M. Visfatin and subclinical atherosclerosis in type 2 diabetes: impact of cardiovascular drugs. Medicina (Kaunas). (2023) 59(7):1324. 10.3390/medicina59071324 37512134 PMC10386106

[82] Du Y Ji Q Cai L Huang F Lai Y Liu Y Association between omentin-1 expression in human epicardial adipose tissue and coronary atherosclerosis. Cardiovasc Diabetol. (2016) 15:90. 10.1186/s12933-016-0406-5 27352781 PMC4924240

[83] Nishimura M Morioka T Hayashi M Kakutani Y Yamazaki Y Kurajoh M Plasma omentin levels are inversely associated with atherosclerosis in type 2 diabetes patients with increased plasma adiponectin levels: a cross-sectional study. Cardiovasc Diabetol. (2019) 18(1):167. 10.1186/s12933-019-0973-3 31805941 PMC6894467

[84] Zhang Y Lv P Li Y Zhang Y Cheng C Hao H Inflammatory cytokine interleukin-6 (IL-6) promotes the proangiogenic ability of adipose stem cells from obese subjects via the IL-6 signaling pathway. Curr Stem Cell Res Ther. (2023) 18(1):93–104. 10.2174/1574888X17666220429103935 36883256

[85] Eswar S Rajagopalan B Ete K Nageswara Rao Gattem S. Serum tumor necrosis factor alpha (TNF-α) levels in obese and overweight adults: correlations with metabolic syndrome and inflammatory markers. Cureus. (2024) 16(7):e64619. 10.7759/cureus.64619 39149648 PMC11326759

[86] Ait-Oufella H Libby P Tedgui A. Anticytokine immune therapy and atherothrombotic cardiovascular risk. Arterioscler Thromb Vasc Biol. (2019) 39(8):1510–9. 10.1161/ATVBAHA.119.311998 31294625 PMC6681658

[87] Geddo F Querio G Asteggiano A Antoniotti S Porcu A Occhipinti A Improving endothelial health with food-derived H(2)S donors: an in vitro study with S-allyl cysteine and with a black-garlic extract enriched in sulfur-containing compounds. Food Funct. (2023) 14(9):4163–72. 10.1039/D3FO00412K 37062967

[88] Wang C Shu L Cheng R Yan M Liang W Zhou J Exercise enhances anti-contractile effects of PVAT through endogenous H_2_S in high-fat diet-induced obesity hypertension. Cardiovasc Drugs Ther. (2024). 10.1007/s10557-024-07612-x 39133260

[89] Oliveira PB Zochio GP Caetano ESP Da Silva MLS Dias-Junior CA. Vasodilator responses of perivascular adipose tissue-derived hydrogen sulfide stimulated with L-cysteine in pregnancy hypertension-induced endothelial dysfunction in rats. Antioxidants (Basel). (2023) 12(11):1919. 10.3390/antiox12111919 38001772 PMC10669374

[90] Lee RM Lu C Su LY Gao YJ. Endothelium-dependent relaxation factor released by perivascular adipose tissue. J Hypertens. (2009) 27(4):782–90. 10.1097/HJH.0b013e328324ed86 19516177

[91] Xia N Horke S Habermeier A Closs EI Reifenberg G Gericke A Uncoupling of endothelial nitric oxide synthase in perivascular adipose tissue of diet-induced obese mice. Arterioscler Thromb Vasc Biol. (2016) 36(1):78–85. 10.1161/ATVBAHA.115.306263 26586660

[92] Virdis A Duranti E Rossi C Dell’agnello U Santini E Anselmino M Tumour necrosis factor-alpha participates on the endothelin-1/nitric oxide imbalance in small arteries from obese patients: role of perivascular adipose tissue. Eur Heart J. (2015) 36(13):784–94. 10.1093/eurheartj/ehu072 24578389

[93] Chen Y Chen C Feng C Tang A Ma Y He X AVE 3085, a novel endothelial nitric oxide synthase enhancer, attenuates cardiac remodeling in mice through the smad signaling pathway. Arch Biochem Biophys. (2015) 570:8–13. 10.1016/j.abb.2015.02.020 25712222

[94] Li X Ballantyne LL Yu Y Funk CD. Perivascular adipose tissue–derived extracellular vesicle miR-221-3p mediates vascular remodeling. FASEB J. (2019) 33(11):12704–22. 10.1096/fj.201901548R 31469602 PMC6902668

[95] Ballasy N Jadli A Edalat P Gomes K Wijesuriya T Belke D Paracrine effects of perivascular adipose tissue on atherogenesis: role of extracellular vesicles-mediated intercellular communications. FASEB J. (2021) 35(Suppl 1):01712. 10.1096/fasebj.2021.35.S1.01712

[96] Sigdel S Udoh G Albalawy R Wang J. Perivascular adipose tissue and perivascular adipose tissue-derived extracellular vesicles: new insights in vascular disease. Cells. (2024) 13(16):1309. 10.3390/cells13161309 39195199 PMC11353161

[97] Chen HH Li HF Tseng TL Lin H. Perivascular adipose tissue and adipocyte-derived exosomal miRNAs maintain vascular homeostasis. Heliyon. (2023) 9(12):e22607. 10.1016/j.heliyon.2023.e22607 38076178 PMC10709399

[98] Liu J Chen Y. Cell-cell crosstalk between fat cells and immune cells. Am J Physiol Endocrinol Metab. (2024) 327(3):E371–83. 10.1152/ajpendo.00024.2024 39082899

[99] Tutunchi H Ostadrahimi A Hosseinzadeh-Attar MJ Miryan M Mobasseri M Ebrahimi-Mameghani M. A systematic review of the association of neuregulin 4, a brown fat-enriched secreted factor, with obesity and related metabolic disturbances. Obes Rev. (2020) 21(2):e12952. 10.1111/obr.12952 31782243

[100] Liu Y Chen M. Neuregulin 4 as a novel adipokine in energy metabolism. Front Physiol. (2022) 13:1106380. 10.3389/fphys.2022.1106380 36703934 PMC9873244

[101] Jeffery E Wing A Holtrup B Sebo Z Kaplan JL Saavedra-Peña R The adipose tissue microenvironment regulates depot-specific adipogenesis in obesity. Cell Metab. (2016) 24(1):142–50. 10.1016/j.cmet.2016.05.012 27320063 PMC4945385

[102] Siang DTC Lim YC Kyaw AMM Win KN Chia SY Degirmenci U The RNA-binding protein HuR is a negative regulator in adipogenesis. Nat Commun. (2020) 11(1):213. 10.1038/s41467-019-14001-8 31924774 PMC6954112

[103] Kumar RK Yang Y Contreras AG Garver H Bhattacharya S Fink GD Phenotypic changes in T cell and macrophage subtypes in perivascular adipose tissues precede high-fat diet-induced hypertension. Front Physiol. (2021) 12:616055. 10.3389/fphys.2021.616055 33815135 PMC8010306

[104] Wang S Cao S Arhatte M Li D Shi Y Kurz S Adipocyte Piezo1 mediates obesogenic adipogenesis through the FGF1/FGFR1 signaling pathway in mice. Nat Commun. (2020) 11(1):2303. 10.1038/s41467-020-16026-w 32385276 PMC7211025

[105] Veerapaneni P Goo B Ahmadieh S Shi H Kim DS Ogbi M Transgenic overexpression of HDAC9 promotes adipocyte hypertrophy, insulin resistance and hepatic steatosis in aging mice. Biomolecules. (2024) 14(4):494. 10.3390/biom14040494 38672510 PMC11048560

[106] Xu H Barnes GT Yang Q Tan G Yang D Chou CJ Chronic inflammation in fat plays a crucial role in the development of obesity-related insulin resistance. J Clin Invest. (2003) 112(12):1821–30. 10.1172/JCI200319451 14679177 PMC296998

[107] Talukdar S Oh DY Bandyopadhyay G Li D Xu J Mcnelis J Neutrophils mediate insulin resistance in mice fed a high-fat diet through secreted elastase. Nat Med. (2012) 18(9):1407–12. 10.1038/nm.2885 22863787 PMC3491143

[108] Soedono S Cho KW. Adipose tissue dendritic cells: critical regulators of obesity-induced inflammation and insulin resistance. Int J Mol Sci. (2021) 22(16):8666. 10.3390/ijms22168666 34445379 PMC8395475

[109] Hirai S Ohyane C Kim Y-I Lin S Goto T Takahashi N Involvement of mast cells in adipose tissue fibrosis. Am J Physiol Endocrinol Metab. (2014) 306(3):E247–55. 10.1152/ajpendo.00056.2013 24326418

[110] Żelechowska P Agier J Kozłowska E Brzezińska-Błaszczyk E. Mast cells participate in chronic low-grade inflammation within adipose tissue. Obes Rev. (2018) 19(5):686–97. 10.1111/obr.12670 29334696

[111] Moussa K Gurung P Adams-Huet B Devaraj S Jialal I. Increased eosinophils in adipose tissue of metabolic syndrome. J Diabetes Complicat. (2019) 33(8):535–8. 10.1016/j.jdiacomp.2019.05.010 31204245

[112] Vohralik EJ Psaila AM Knights AJ Quinlan KGR. EoTHINophils: eosinophils as key players in adipose tissue homeostasis. Clin Exp Pharmacol Physiol. (2020) 47(8):1495–505. 10.1111/1440-1681.13304 32163614

[113] Nishimura S Manabe I Takaki S Nagasaki M Otsu M Yamashita H Adipose natural regulatory B cells negatively control adipose tissue inflammation. Cell Metab. (2013) 18(5):759–66. 10.1016/j.cmet.2013.09.017 24209772

[114] Srikakulapu P Upadhye A Rosenfeld SM Marshall MA Mcskimming C Hickman AW Perivascular adipose tissue harbors atheroprotective IgM-producing B cells. Front Physiol. (2017) 8:719. 10.3389/fphys.2017.00719 28970806 PMC5609437

[115] Yu Y Bai H Wu F Chen J Li B Li Y. Tissue adaptation of regulatory T cells in adipose tissue. Eur J Immunol. (2022) 52(12):1898–908. 10.1002/eji.202149527 36369886

[116] Jacks RD Lumeng CN. Macrophage and T cell networks in adipose tissue. Nat Rev Endocrinol. (2024) 20(1):50–61. 10.1038/s41574-023-00908-2 37872302

[117] Nishimura S Manabe I Nagasaki M Eto K Yamashita H Ohsugi M CD8 + effector T cells contribute to macrophage recruitment and adipose tissue inflammation in obesity. Nat Med. (2009) 15(8):914–20. 10.1038/nm.1964 19633658

[118] Dos Reis Costa DEF de Araújo NF Nóbrega NRC de Assis Rabelo Ribeiro N de Oliveira ACC Dos Santos Aggum Capettini L Contribution of RAS, ROS and COX-1-derived prostanoids to the contractile profile of perivascular adipose tissue in cafeteria diet-induced obesity. Life Sci. (2022) 309:120994. 10.1016/j.lfs.2022.120994 36155180

[119] Das E Moon JH Lee JH Thakkar N Pausova Z Sung HK. Adipose tissue and modulation of hypertension. Curr Hypertens Rep. (2018) 20(11):96. 10.1007/s11906-018-0894-7 30229358

[120] Pritchard KA Groszek L Smalley DM Sessa WC Wu M Villalon P Native low-density lipoprotein increases endothelial cell nitric oxide synthase generation of superoxide anion. Circ Res. (1995) 77(3):510–8. 10.1161/01.RES.77.3.510 7543827

[121] Ma Y Li L Shao Y Bai X Bai T Huang X. Methotrexate improves perivascular adipose tissue/endothelial dysfunction via activation of AMPK/eNOS pathway. Mol Med Rep. (2017) 15(4):2353–9. 10.3892/mmr.2017.6225 28259947

[122] Tai GJ Ma YJ Feng JL Li JP Qiu S Yu QQ NLRP3 inflammasome-mediated premature immunosenescence drives diabetic vascular aging dependent on the induction of perivascular adipose tissue dysfunction. Cardiovasc Res. (2024) 121(1):77–96. 10.1093/cvr/cvae07 38643484

[123] Mitri J Tomah S Furtado J Tasabehji MW Hamdy O. Plasma free fatty acids and metabolic effect in type 2 diabetes, an ancillary study from a randomized clinical trial. Nutrients. (2021) 13(4):1145. 10.3390/nu13041145 33807135 PMC8065525

[124] Tang YH Wang YH Chen CC Chan CJ Tsai FJ Chen SY. Genetic and functional effects of adiponectin in type 2 diabetes mellitus development. Int J Mol Sci. (2022) 23(21):13544. 10.3390/ijms232113544 36362336 PMC9658884

[125] Bako HY Ibrahim MA Isah MS Ibrahim S. Inhibition of JAK-STAT and NF-κB signalling systems could be a novel therapeutic target against insulin resistance and type 2 diabetes. Life Sci. (2019) 239:117045. 10.1016/j.lfs.2019.117045 31730866

[126] Seo JB Riopel M Cabrales P Huh JY Bandyopadhyay GK Andreyev AY Knockdown of Ant2 reduces adipocyte hypoxia and improves insulin resistance in obesity. Nat Metab. (2019) 1(1):86–97. 10.1038/s42255-018-0003-x 31528845 PMC6746433

[127] Yalçın T Oğuz SH Bayraktar M Rakıcıoğlu N. Anthropometric measurements and serum TNF-α, IL-6 and adiponectin in type 2 diabetes. Diabetol Int. (2022) 13(2):396–406. 10.1007/s13340-021-00553-y 35463864 PMC8980124

[128] Sprague AH Khalil RA. Inflammatory cytokines in vascular dysfunction and vascular disease. Biochem Pharmacol. (2009) 78(6):539–52. 10.1016/j.bcp.2009.04.029 19413999 PMC2730638

[129] Hattori Y Suzuki K Hattori S Kasai K. Metformin inhibits cytokine-induced nuclear factor kappaB activation via AMP-activated protein kinase activation in vascular endothelial cells. Hypertension. (2006) 47(6):1183–8. 10.1161/01.HYP.0000221429.94591.72 16636195

[130] Li M Van Esch B Henricks PAJ Folkerts G Garssen J. The anti-inflammatory effects of short chain fatty acids on lipopolysaccharide- or tumor necrosis factor α-stimulated endothelial cells via activation of GPR41/43 and inhibition of HDACs. Front Pharmacol. (2018) 9:533. 10.3389/fphar.2018.00533 29875665 PMC5974203

[131] Miao CY Li ZY. The role of perivascular adipose tissue in vascular smooth muscle cell growth. Br J Pharmacol. (2012) 165(3):643–58. 10.1111/j.1476-5381.2011.01404.x 21470202 PMC3315037

[132] Haghikia A Landmesser U. Lipoproteins and cardiovascular redox signaling: role in atherosclerosis and coronary disease. Antioxid Redox Signal. (2018) 29(3):337–52. 10.1089/ars.2017.7052 28817963

[133] McCarthy CG Martinez-Quinones P Klee NS Watts SW Calmasini FB Komic A Hypertension induced morphological and physiological changes in cells of the arterial wall. Am J Hypertens. (2018) 31(10):1067–78. 10.1093/ajh/hpy083 29788246 PMC6132119

[134] Song Y Jia H Hua Y Wu C Li S Li K The molecular mechanism of aerobic exercise improving vascular remodeling in hypertension. Front Physiol. (2022) 13:792292. 10.3389/fphys.2022.792292 35295586 PMC8919036

[135] Persson P Marchetti M Friederich-Persson M. Browning of perivascular adipose tissue prevents vascular dysfunction and reduces hypertension in angiotensin II-infused mice. Am J Physiol Regul Integr Comp Physiol. (2023) 325(3):R290–8. 10.1152/ajpregu.00043.2023 37458378

[136] Cerecedo D Martínez-Vieyra I Hernández-Rojo I Hernández-Cruz A Rincón-Heredia R Millán-Aldaco D Reactive oxygen species downregulate dystroglycans in the megakaryocytes of rats with arterial hypertension. Exp Cell Res. (2023) 433(2):113847. 10.1016/j.yexcr.2023.113847 37931771

[137] Abais-Battad JM Lund H Dasinger JH Fehrenbach DJ Cowley AW Mattson DL. NOX2-derived reactive oxygen species in immune cells exacerbates salt-sensitive hypertension. Free Radic Biol Med. (2020) 146:333–9. 10.1016/j.freeradbiomed.2019.11.014 31730933 PMC6942201

[138] Mikolajczyk TP Nosalski R Skiba DS Koziol J Mazur M Justo-Junior AS 1,2,3,4,6-Penta-O-galloyl-β-d-glucose modulates perivascular inflammation and prevents vascular dysfunction in angiotensin II-induced hypertension. Br J Pharmacol. (2019) 176(12):1951–65. 10.1111/bph.14583 30658013 PMC6534792

[139] Sena CM Pereira A Fernandes R Letra L Seiça RM. Adiponectin improves endothelial function in mesenteric arteries of rats fed a high-fat diet: role of perivascular adipose tissue. Br J Pharmacol. (2017) 174(20):3514–26. 10.1111/bph.13756 28236429 PMC5610162

[140] Itani HA Mcmaster WG Jr Saleh MA Nazarewicz RR Mikolajczyk TP Kaszuba AM Activation of human T cells in hypertension: studies of humanized mice and hypertensive humans. Hypertension. (2016) 68(1):123–32. 10.1161/HYPERTENSIONAHA.116.07237 27217403 PMC4900908

[141] Oikonomou EK Marwan M Desai MY Mancio J Alashi A Hutt Centeno E Non-invasive detection of coronary inflammation using computed tomography and prediction of residual cardiovascular risk (the CRISP CT study): a post-hoc analysis of prospective outcome data. Lancet. (2018) 392(10151):929–39. 10.1016/S0140-6736(18)31114-0 30170852 PMC6137540

[142] Antoniades C Antonopoulos AS Deanfield J. Imaging residual inflammatory cardiovascular risk. Eur Heart J. (2020) 41(6):748–58. 10.1093/eurheartj/ehz474 31317172

[143] Oikonomou EK Williams MC Kotanidis CP Desai MY Marwan M Antonopoulos AS A novel machine learning-derived radiotranscriptomic signature of perivascular fat improves cardiac risk prediction using coronary CT angiography. Eur Heart J. (2019) 40(43):3529–43. 10.1093/eurheartj/ehz592 31504423 PMC6855141

[144] Chan K Wahome E Tsiachristas A Antonopoulos AS Patel P Lyasheva M Inflammatory risk and cardiovascular events in patients without obstructive coronary artery disease: the ORFAN multicentre, longitudinal cohort study. Lancet. (2024) 403(10444):2606–18. 10.1016/S0140-6736(24)00596-8 38823406 PMC11664027

[145] Ardissino M Mccracken C Bard A Antoniades C Neubauer S Harvey NC Pericardial adiposity is independently linked to adverse cardiovascular phenotypes: a CMR study of 42 598 UK biobank participants. Eur Heart J Cardiovasc Imaging. (2022) 23(11):1471–81. 10.1093/ehjci/jeac101 35640889 PMC9584621

[146] Antonopoulos AS Angelopoulos A Papanikolaou P Simantiris S Oikonomou EK Vamvakaris K Biomarkers of vascular inflammation for cardiovascular risk prognostication: a meta-analysis. JACC Cardiovasc Imaging. (2022) 15(3):460–71. 10.1016/j.jcmg.2021.09.014 34801448

[147] Vasques ACJ Souza JR Yamanaka A De Oliveira Mda S Novaes FS Pareja JC Sagittal abdominal diameter as a marker for epicardial adipose tissue in premenopausal women. Metab Clin Exp. (2013) 62(7):1032–6. 10.1016/j.metabol.2013.01.022 23540720

[148] Gaborit B Julla JB Fournel J Ancel P Soghomonian A Deprade C Fully automated epicardial adipose tissue volume quantification with deep learning and relationship with CAC score and micro/macrovascular complications in people living with type 2 diabetes: the multicenter EPIDIAB study. Cardiovasc Diabetol. (2024) 23(1):328. 10.1186/s12933-024-02411-y 39227844 PMC11373274

[149] Li Y Zhang W Hu Y Xu Z Huo Q Qi H Pericoronary adipose tissue radiomics features as imaging markers for coronary artery disease risk assessment: insights from gene expression analysis. Cardiovasc Diabetol. (2024) 23(1):444. 10.1186/s12933-024-02530-6 39696214 PMC11657505

[150] Su X Peng D. Emerging functions of adipokines in linking the development of obesity and cardiovascular diseases. Mol Biol Rep. (2020) 47(10):7991–8006. 10.1007/s11033-020-05732-9 32888125

[151] Qi L Doria A Manson JAE Meigs JB Hunter D Mantzoros CS Adiponectin genetic variability, plasma adiponectin, and cardiovascular risk in patients with type 2 diabetes. Diabetes. (2006) 55(5):1512–6. 10.2337/db05-1520 16644713

[152] Guzik TJ Nosalski R Maffia P Drummond GR. Immune and inflammatory mechanisms in hypertension. Nat Rev Cardiol. (2024) 21(6):396–416. 10.1038/s41569-023-00964-1 38172242

[153] Lingvay I Cohen RV Roux CWL Sumithran P. Obesity in adults. Lancet. (2024) 404(10456):972–87. 10.1016/S0140-6736(24)01210-8 39159652

[154] Davis CL Lorig K. Lifestyle interventions for obesity. JAMA. (2024) 332(17):1488. 10.1001/jama.2024.17533 39382884

[155] Elmaleh-Sachs A Schwartz JL Bramante CT Nicklas JM Gudzune KA Jay M. Obesity management in adults: a review. JAMA. (2023) 330(20):2000–15. 10.1001/jama.2023.19897 38015216 PMC11325826

[156] Khoo J Dhamodaran S Chen DD Yap SY Chen RY Tian RH. Exercise-induced weight loss is more effective than dieting for improving adipokine profile, insulin resistance, and inflammation in obese men. Int J Sport Nutr Exerc Metab. (2015) 25(6):566–75. 10.1123/ijsnem.2015-0025 26011919

[157] Saxton SN Toms LK Aldous RG Withers SB Ohanian J Heagerty AM. Restoring perivascular adipose tissue function in obesity using exercise. Cardiovasc Drugs Ther. (2021) 35(6):1291–304. 10.1007/s10557-020-07136-0 33687595 PMC8578065

[158] Guo P Zhou Y Zhu Y. Effects of a school-based lifestyle intervention on ideal cardiovascular health in Chinese children and adolescents: a national, multicentre, cluster-randomised controlled trial. Lancet Glob Health. (2023) 11(Suppl 1):S14. 10.1016/S2214-109X(23)00097-9 36866471

[159] Li M Li J Xu Y Gao J Cao Q Ding Y Effect of 5:2 regimens: energy-restricted diet or low-volume high-intensity interval training combined with resistance exercise on glycemic control and cardiometabolic health in adults with overweight/obesity and type 2 diabetes: a three-arm randomized controlled trial. Diabetes Care. (2024) 47(6):1074–83. 10.2337/dc24-0241 38638032 PMC11116924

[160] Woo KS Chook P Yu CW Sung RYT Qiao M Leung SSF Effects of diet and exercise on obesity-related vascular dysfunction in children. Circulation. (2004) 109(16):1981–6. 10.1161/01.CIR.0000126599.47470.BE 15066949

[161] Kusminski CM Bickel PE Scherer PE. Targeting adipose tissue in the treatment of obesity-associated diabetes. Nat Rev Drug Discov. (2016) 15(9):639–60. 10.1038/nrd.2016.75 27256476

[162] Ussher JR Drucker DJ. Glucagon-like peptide 1 receptor agonists: cardiovascular benefits and mechanisms of action. Nat Rev Cardiol. (2023) 20(7):463–74. 10.1038/s41569-023-00849-3 36977782

[163] Szekeres Z Nagy A Jahner K Szabados E. Impact of selected glucagon-like peptide-1 receptor agonists on Serum lipids, adipose tissue, and muscle metabolism-a narrative review. Int J Mol Sci. (2024) 25(15):8214. 10.3390/ijms25158214 39125786 PMC11311305

[164] Meier JJ. GLP-1 receptor agonists for individualized treatment of type 2 diabetes mellitus. Nat Rev Endocrinol. (2012) 8(12):728–42. 10.1038/nrendo.2012.140 22945360

[165] Delessa Challa T Beaton N Arnold M Rudofsky G Langhans W Wolfrum C. Regulation of adipocyte formation by GLP-1/GLP-1R signaling. J Biol Chem. (2012) 287(9):6421–30. 10.1074/jbc.M111.310342 22207759 PMC3307265

[166] Le Kim Chung T Yoshida M Hosaka T Sakaue H Harada N Sakai T Exendin-4, a GLP-1 receptor agonist, directly induces adiponectin expression through protein kinase A pathway and prevents inflammatory adipokine expression. Biochem Biophys Res Commun. (2009) 390(3):613–8. 10.1016/j.bbrc.2009.10.015 19850014

[167] Fujiwara Y Shiraishi D Mizuta H Komohara Y Takeya M. Glucagon-like peptide-1 (GLP-1) induces M2 polarization of human macrophages via STAT3 activation. Biochem Biophys Res Commun. (2012) 425(2):304–8. 10.1016/j.bbrc.2012.07.086 22842565

[168] Marso SP Daniels GH Brown-Frandsen K Kristensen P Mann JF Nauck MA Liraglutide and cardiovascular outcomes in type 2 diabetes. N Engl J Med. (2016) 375(4):311–22. 10.1056/NEJMoa1603827 27295427 PMC4985288

[169] Santilli F Simeone PG Guagnano MT Leo M Maccarone MT Di Castelnuovo A Effects of liraglutide on weight loss, fat distribution, and β-cell function in obese subjects with prediabetes or early type 2 diabetes. Diabetes Care. (2017) 40(11):1556–64. 10.2337/dc17-0589 28912305

[170] Verma S Bhatt DL Bain SC Buse JB Mann JFE Marso SP Effect of liraglutide on cardiovascular events in patients with type 2 diabetes mellitus and polyvascular disease: results of the LEADER trial. Circulation. (2018) 137(20):2179–83. 10.1161/CIRCULATIONAHA.118.033898 29760228

[171] Que Y Shi J Zhang Z Sun L Li H Qin X Ion cocktail therapy for myocardial infarction by synergistic regulation of both structural and electrical remodeling. Exploration (Beijing). (2024) 4(3):20230067. 10.1002/EXP.20230067 38939858 PMC11189571

[172] Zelniker TA Wiviott SD Raz I Im K Goodrich EL Bonaca MP SGLT2 inhibitors for primary and secondary prevention of cardiovascular and renal outcomes in type 2 diabetes: a systematic review and meta-analysis of cardiovascular outcome trials. Lancet. (2019) 393(10166):31–9. 10.1016/S0140-6736(18)32590-X 30424892

[173] Xu L Ota T. Emerging roles of SGLT2 inhibitors in obesity and insulin resistance: focus on fat browning and macrophage polarization. Adipocyte. (2018) 7(2):121–8. 10.1080/21623945.2017.1413516 29376471 PMC6152529

[174] Xu L Nagata N Nagashimada M Zhuge F Ni Y Chen G SGLT2 inhibition by empagliflozin promotes fat utilization and browning and attenuates inflammation and insulin resistance by polarizing M2 macrophages in diet-induced obese mice. EBioMedicine. (2017) 20:137–49. 10.1016/j.ebiom.2017.05.028 28579299 PMC5478253

[175] Zinman B Wanner C Lachin JM Fitchett D Bluhmki E Hantel S Empagliflozin, cardiovascular outcomes, and mortality in type 2 diabetes. N Engl J Med. (2015) 373(22):2117–28. 10.1056/NEJMoa1504720 26378978

[176] Wanner C Lachin JM Inzucchi SE Fitchett D Mattheus M George J Empagliflozin and clinical outcomes in patients with type 2 diabetes mellitus, established cardiovascular disease, and chronic kidney disease. Circulation. (2018) 137(2):119–29. 10.1161/CIRCULATIONAHA.117.028268 28904068

[177] Šatný M Jaroslav H Vrablík M. Statins and inflammation. Current atherosclerosis reports. (2021). 10.1007/s11883-021-00977-6 34851454

[178] Sanna F Margaritis M Antoniades C. Perivascular adipose tissue as an endocrine organ: the role of statins. Curr Pharm Des. (2017). 10.2174/1381612823666170926133843 28950824

[179] Guri AJ Hontecillas R Bassaganya-Riera J. Abscisic acid synergizes with rosiglitazone to improve glucose tolerance and down-modulate macrophage accumulation in adipose tissue: possible action of the cAMP/PKA/PPAR γ axis. Clin Nutr. (2010) 29(5):646–53. 10.1016/j.clnu.2010.02.003 20207056 PMC2888662

[180] Derosa G Maffioli P. Peroxisome proliferator-activated receptor-γ (PPAR-γ) agonists on glycemic control, lipid profile and cardiovascular risk. Curr Mol Pharmacol. (2012) 5(2):272–81. 10.2174/1874467211205020272 22122457

[181] Bajzer M Olivieri M Haas MK Pfluger PT Magrisso IJ Foster MT Cannabinoid receptor 1 (CB1) antagonism enhances glucose utilisation and activates brown adipose tissue in diet-induced obese mice. Diabetologia. (2011) 54(12):3121–31. 10.1007/s00125-011-2302-6 21987346 PMC8464406

[182] Han JH Shin H Rho JG Kim JE Son DH Yoon J Peripheral cannabinoid 1 receptor blockade mitigates adipose tissue inflammation via NLRP3 inflammasome in mouse models of obesity. Diabetes Obes Metab. (2018) 20(9):2179–89. 10.1111/dom.13350 29740969

[183] Epstein M. Reduction of cardiovascular risk in chronic kidney disease by mineralocorticoid receptor antagonism. Lancet Diabetes Endocrinol. (2015) 3(12):993–1003. 10.1016/S2213-8587(15)00289-2 26429402

[184] Guo C Ricchiuti V Lian BQ Yao TM Coutinho P Romero JR Mineralocorticoid receptor blockade reverses obesity-related changes in expression of adiponectin, peroxisome proliferator-activated receptor-gamma, and proinflammatory adipokines. Circulation. (2008) 117(17):2253–61. 10.1161/CIRCULATIONAHA.107.748640 18427128 PMC2746647

[185] Aminian A Zajichek A Arterburn DE Wolski KE Brethauer SA Schauer PR Association of metabolic surgery with major adverse cardiovascular outcomes in patients with type 2 diabetes and obesity. JAMA. (2019) 322(13):1271–82. 10.1001/jama.2019.14231 31475297 PMC6724187

[186] Aghamohammadzadeh R Greenstein AS Yadav R Jeziorska M Hama S Soltani F Effects of bariatric surgery on human small artery function: evidence for reduction in perivascular adipocyte inflammation, and the restoration of normal anticontractile activity despite persistent obesity. J Am Coll Cardiol. (2013) 62(2):128–35. 10.1016/j.jacc.2013.04.027 23665100 PMC3791397

[187] Klein S Fontana L Young VL Coggan AR Kilo C Patterson BW Absence of an effect of liposuction on insulin action and risk factors for coronary heart disease. N Engl J Med. (2004) 350(25):2549–57. 10.1056/NEJMoa033179 15201411

[188] Shen J Obin MS Zhao L. The gut microbiota, obesity and insulin resistance. Mol Aspects Med. (2013) 34(1):39–58. 10.1016/j.mam.2012.11.001 23159341

[189] Zhao LY Mei JX Yu G Lei L Zhang WH Liu K Role of the gut microbiota in anticancer therapy: from molecular mechanisms to clinical applications. Signal Transduct Target Ther. (2023) 8(1):201. 10.1038/s41392-023-01406-7 37179402 PMC10183032

[190] Li Z Xiong W Liang Z Wang J Zeng Z Kołat D Critical role of the gut microbiota in immune responses and cancer immunotherapy. J Hematol Oncol. (2024) 17(1):33. 10.1186/s13045-024-01541-w 38745196 PMC11094969

[191] Chen L Ishigami T Nakashima-Sasaki R Kino T Doi H Minegishi S Commensal microbe-specific activation of B2 cell subsets contributes to atherosclerosis development independently of lipid metabolism. EBioMedicine. (2016) 13:237–47. 10.1016/j.ebiom.2016.10.030 27810309 PMC5264349

[192] Zhang ZB Cheng YW Xu L Li JQ Pan X Zhu M Activation of β3-adrenergic receptor by mirabegron prevents aortic dissection/aneurysm by promoting lymphangiogenesis in perivascular adipose tissue. Cardiovasc Res. (2024) 120(17):2307–19. 10.1093/cvr/cvae213 39288197

[193] Chiabrando F Lanzillotta M Palumbo D Pedica F Caruso M Capurso G Treating type 2 autoimmune pancreatitis with colchicine: a case series. Ann Intern Med. (2021) 174(12):1775–6. 10.7326/L21-0281 34633831

[194] Deftereos SG Beerkens FJ Shah B Giannopoulos G Vrachatis DA Giotaki SG Colchicine in cardiovascular disease: in-depth review. Circulation. (2022) 145(1):61–78. 10.1161/CIRCULATIONAHA.121.056171 34965168 PMC8726640

[195] Nidorf SM Ben-Chetrit E Ridker PM. Low-dose colchicine for atherosclerosis: long-term safety. Eur Heart J. (2024) 45(18):1596–601. 10.1093/eurheartj/ehae208 38596868

[196] Gollasch M. Adipose-vascular coupling and potential therapeutics. Annu Rev Pharmacol Toxicol. (2017) 57:417–36. 10.1146/annurev-pharmtox-010716-104542 27732801

[197] Lee YC Chang HH Chiang CL Liu CH Yeh JI Chen MF Role of perivascular adipose tissue-derived methyl palmitate in vascular tone regulation and pathogenesis of hypertension. Circulation. (2011) 124(10):1160–71. 10.1161/CIRCULATIONAHA.111.027375 21844078

[198] Rendon CJ Sempere L Lauver A Watts SW Contreras GA. Anatomical location, sex, and age modulate adipocyte progenitor populations in perivascular adipose tissues. Front Physiol. (2024) 15:1411218. 10.3389/fphys.2024.1411218 39072214 PMC11282503

[199] Victorio JA Da Costa RM Tostes RC Davel AP. Modulation of vascular function by perivascular adipose tissue: sex differences. Curr Pharm Des. (2020) 26(30):3768–77. 10.2174/1381612826666200701211912 32611295

[200] Somani YB Pawelczyk JA De Souza MJ Kris-Etherton PM Proctor DN. Aging women and their endothelium: probing the relative role of estrogen on vasodilator function. Am J Physiol Heart Circ Physiol. (2019) 317(2):H395–404. 10.1152/ajpheart.00430.2018 31173499 PMC6732482

[201] Tankó LB Christiansen C. Adipose tissue, insulin resistance and low-grade inflammation: implications for atherogenesis and the cardiovascular harm of estrogen plus progestogen therapy. Climacteric. (2006) 9(3):169–80. 10.1080/13697130600738765 16766431

[202] Chen Y Qin Z Wang Y Li X Zheng Y Liu Y. Role of inflammation in vascular disease-related perivascular adipose tissue dysfunction. Front Endocrinol (Lausanne). (2021) 12:710842. 10.3389/fendo.2021.710842 34456867 PMC8385491

[203] Luo YL Xu CF Li HJ Cao ZT Liu J Wang JL Macrophage-specific in vivo gene editing using cationic lipid-assisted polymeric nanoparticles. ACS Nano. (2018) 12(2):994–1005. 10.1021/acsnano.7b07874 29314827

[204] Schmidt R Steinhart Z Layeghi M Freimer JW Bueno R Nguyen VQ CRISPR activation and interference screens decode stimulation responses in primary human T cells. Science. (2022) 375(6580):eabj4008. 10.1126/science.abj4008 35113687 PMC9307090

